# The effect of fatty diacid acylation of human PYY_3-36_ on Y_2_ receptor potency and half-life in minipigs

**DOI:** 10.1038/s41598-021-00654-3

**Published:** 2021-10-27

**Authors:** Søren Østergaard, Johan F. Paulsson, Jacob Kofoed, Franziska Zosel, Jørgen Olsen, Claus Bekker Jeppesen, Jane Spetzler, Lars Ynddal, Luise Gram Schleiss, Berit Østergaard Christoffersen, Kirsten Raun, Ulrich Sensfuss, Flemming Seier Nielsen, Rasmus Jørgensen, Birgitte S. Wulff

**Affiliations:** 1 Global Research Technologies, Novo Nordisk A/S, Novo Nordisk Research Park, 2760 Maaloev, Denmark; 2 Present Address: Gubra Aps, Hørsholm Kongevej 11B, 2970 Hørsholm, Denmark; 3 Present Address: STipe Therapeutics, Copenhagen, Denmark; 4 Present Address: CitoKi Pharma, Værløse, Denmark

**Keywords:** Lead optimization, Pharmacology, Drug discovery, Drug delivery, Medicinal chemistry, Pharmacology, Lipopeptides, Biochemistry, Peptides

## Abstract

Peptides are notoriously known to display very short *in vivo* half-lives often measured in minutes which in many cases greatly reduces or eliminates sufficient *in vivo* efficacy. To obtain long half-lives allowing for up to once-weekly dosing regimen, fatty acid acylation (lipidation) have been used to non-covalently associate the peptide to serum albumin thus serving as a circulating depot. This approach is generally considered in the scientific and patent community as a standard approach to protract almost any given peptide. However, it is not trivial to prolong the half-life of peptides by lipidation and still maintain high potency and good formulation properties. Here we show that attaching a fatty acid to the obesity-drug relevant peptide PYY_3-36_ is not sufficient for long pharmacokinetics (PK), since the position in the backbone, but also type of fatty acid and linker strongly influences PK and potency. Furthermore, understanding the proteolytic stability of the backbone is key to obtain long half-lives by lipidation, since backbone cleavage still occurs while associated to albumin. Having identified a PYY analogue with a sufficient half-life, we show that in combination with a GLP-1 analogue, liraglutide, additional weight loss can be achieved in the obese minipig model.

## Introduction

Peptide YY (PYY_1-36_) is a 36 amino acid peptide that is co-released with GLP-1 from the L-cells in the distal gut in response to ingestion of nutrients^1,2^ and is part of a family of peptides including neuropeptide Y (NPY) and pancreatic polypeptide (PP), which all share a common structural fold known as the PP-fold^3^. All three peptides bind to the four receptors Y_1_ (Y_1_R), Y_2_ (Y_2_R), Y_4_ (Y_4_R) and Y_5_ (Y_5_R), classified as the NPY receptor family. While PYY and NPY predominantly bind to the Y_1_R, Y_2_R and Y_5_R with nanomolar affinity, PP mainly targets the Y_4_R. Common to all three peptides is the binding primarily of the C-terminus into a deep groove in the receptor^4–6^. Peptide YY is released as PYY_1-36_ but is readily cleaved to the more Y_2_R selective PYY_3-36_ by DPPIV^7^, which was thought to be the major circulating form of PYY. Recently, however, C-terminally truncated and inactive of versions PYY_1-34_ and PYY_3-34_ have been identified and they may, in fact, be more abundant in plasma than the active versions^8–10^.

PYY_3-36_ has been extensively characterized with respect to promotion of satiety and regulation of energy balance either as standalone treatment or in combination with GLP-1^1,11–15^. Consequently, PYY_3-36_ administration has attracted attention as a novel anti-obesity treatment. The half-life of PYY_3-36_ is very short below 10 minutes in plasma, due to rapid renal clearance and enzymatic degradation^8,16^. As a result, continuous administration, e.g. by pumps, has been used to report satiety effects in humans. More attractive from a pharmaceutical point of view, however, is the design of a PYY_3-36_ analogue displaying a much-prolonged half-life after administration by s.c. injection.

**Figure 1:**
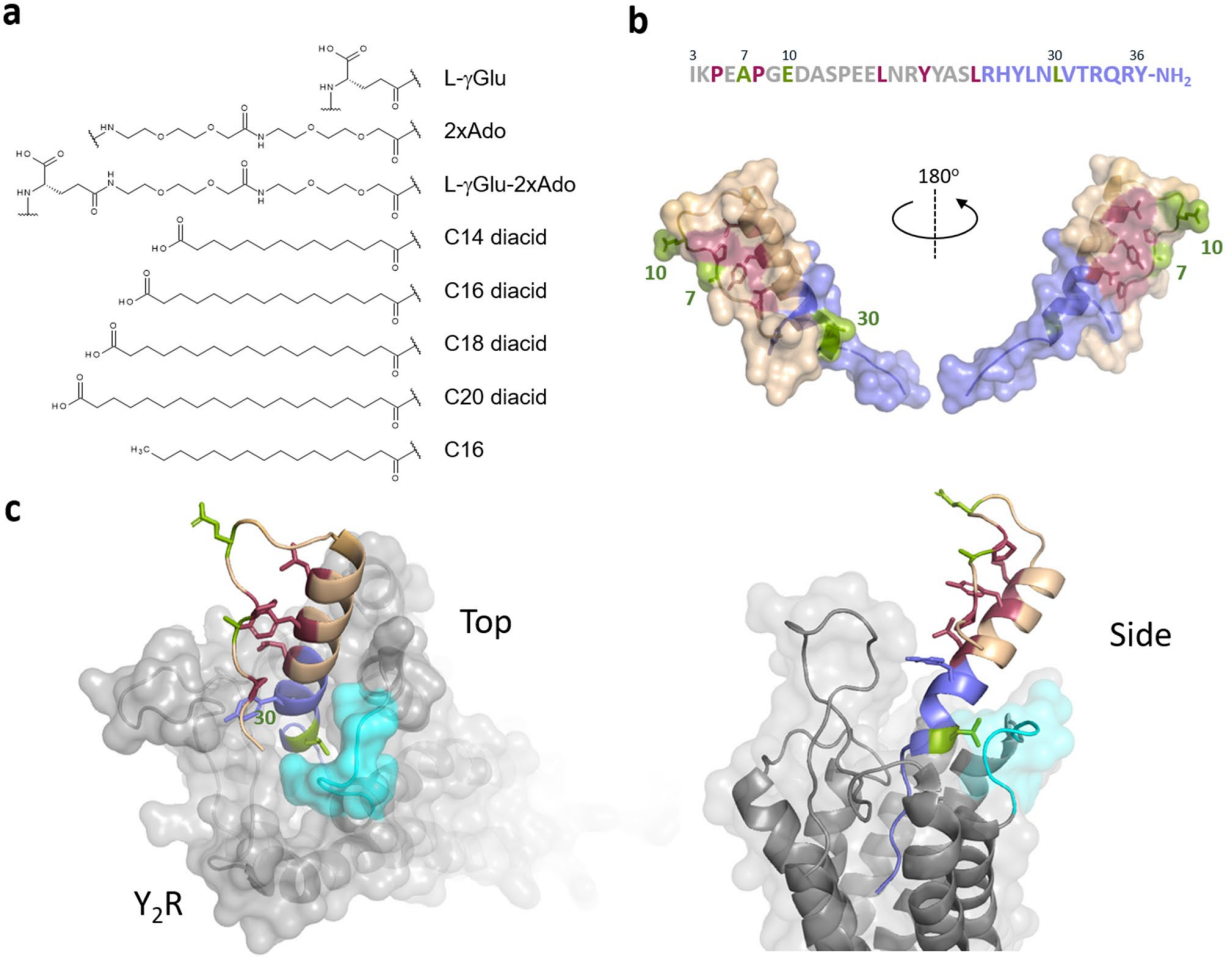
An overview of protraction moieties and PYY_3-36_ structure activity relationship with respect to Y_2_R interaction. (**a**) Structure of fatty acids and diacids, linker and spacer used in the lipidation scan of PYY_3-36_. (**b**) Sequence of PYY_3-36_ and structure of PYY_3-36_ (pdb:2DEZ). Depicted in magenta are core residues; shown in blue are critical residues for the interaction interacting with the Y_2_R. (**c**) In a model of PYY_3-36_ with Y_2_R (grey) a loop comprising the receptor residues 294-299 (cyan) is proposed to in proximity with position 30 (model coordinator from reference^33^.

In order to avoid renal clearance of PYY_3-36_, approaches have been reported that increase the hydrodynamic volume of PYY_3-36_ by conjugation of polyethylene glycol (PEG) to PYY_3-36_ and analogues thereof^17,18^, conjugation to serum albumin^19,20^ or immunoglobulin Fc domain^21^. These methods have been shown to prolong the half-life while maintaining the pharmacological activity. Also sustained release of a PYY analogue has been reported and shown effect in a human trial study^22^. Serum albumin and the Fc domain of antibodies display a very long half-life of approximately 19 days in humans due to recycling via the neonatal Fc receptor (FcRn)^23^. Albumin is also known as a transporter and binder of fatty acids and other hydrophobic small molecule drugs. Consequently, prolonged half-lives can be achieved by attaching a fatty acid to a target protein or peptide, which, due to the binding of the fatty acid to albumin, then serves as a circulating depot of the target protein or peptide. This has been shown to be an attractive way of prolonging the *in vivo* effect of the insulin analogues determir and degludec^24–26^, as well as GLP-1 analogues^27,28^. In the once-daily dosed GLP-1 analogue, liraglutide, a palmitic acid (C16) is connected to a l-γ-glutamyl (γGlu) residue on Lys^26^, whereas in the once-weekly dosed GLP-1 analogue, semaglutide^28^, it is an octadecanedioic acid (C18 diacid) linked to a γGlu moiety and connected to a spacer, consisting of two 8-amino-3,6-dioxaoctanoic acid (Ado) units attached to the side chain of Lys^26^ (Fig. 1a). The C18 diacid has also been attached to PYY_3-36_ as a stabling moiety between positions 10 and 17 or 23 and 30, showing extended half-life in a rodent model^29^. In the once-weekly dosed GLP-1/GIP dual acting analogue, tirzepatide, the protraction is mediated by eicosanedioic acid (C20 diacid) linked to γGlu and a 2xAdo unit, which is then attached to the side chain of a lysine residue^30^. For liraglutide and the insulin analogue degludec, the prolonged half-life is also achieved by self-association at the site of injection in addition to albumin binding^26,31^, whereas in semaglutide and tirzepatide much stronger albumin binding is the main driver of protraction. A tight association to albumin has been considered essential to obtain very long half-lives, necessary for obtaining a once-weekly dosing profile.

We synthesized a large series of PYY_3-36_ analogues (Table 1) in which we studied the half-life in minipigs, the NPY receptor potencies and binding properties for both human and porcine albumin as a function of fatty diacid derivatization. This was achieved by synthesizing a series of analogues of PYY_3-36_ where the fatty diacid side chain from semaglutide (C18 diacid-γGlu-2xAdo) was positioned on the side chain of a lysine residue throughout the backbone of PYY_3-36_. The *in vitro* potency on Y_1_R, Y_2_R and Y_4_R as a function of fatty diacid position was also determined, as well as binding to the Y_2_R in the absence and presence of albumin for a selected set of analogues. Analogues with the fatty diacid protractor in position 30 were also studied with respect to length of fatty diacid and linker type, as well as backbone modifications and the impact these modifications may have on the half-life and potency. Finally, we performed an *in vivo* metabolism study in the obese minipig model with one of the optimized analogues in combination with the once-daily GLP-1 analogue, liraglutide.

**Table 1:** Overview of the fatty diacid protracted PYY analogues and their half-lives in minipigs.

No	Backbone modification	Pos.	Fatty acid	Linker	Half-life (hours)	No.	Backbone modification	Pos.	Fatty acid	Linker	Half-life (hours)
**1**	none	N^α^	C18 diacid	γGlu-2xAdo	17	**32**	Lys^34^	34	C18 diacid	γGlu-2xAdo	30
**2**	none	4	C18 diacid	γGlu-2xAdo	14	**33**	Lys^35^	35	C18 diacid	γGlu-2xAdo	67
**3**	Lys^5^	5	C18 diacid	γGlu-2xAdo	8.8	**34**	Lys^30^	30	C14 diacid	γGlu-2xAdo	4
**4**	Lys^6^	6	C18 diacid	γGlu-2xAdo	7.2	**35**	Lys^30^	30	C16 diacid	γGlu-2xAdo	28
**5**	Lys^7^	7	C18 diacid	γGlu-2xAdo	11	**36**	Lys^30^	30	C20 diacid	γGlu-2xAdo	99
**6**	Lys^8^	8	C18 diacid	γGlu-2xAdo	5.9	**37**	Lys^30^	30	C14 diacid	2xAdo	2
**7**	Lys^9^	9	C18 diacid	γGlu-2xAdo	12	**38**	Lys^30^	30	C16 diacid	2xAdo	4
**8**	Lys^10^	10	C18 diacid	γGlu-2xAdo	8.4	**39**	Lys^30^	30	C18 diacid	2xAdo	13
**9**	Lys^11^	11	C18 diacid	γGlu-2xAdo	11	**40**	Lys^30^	30	C20 diacid	2xAdo	20
**10**	Lys^12^	12	C18 diacid	γGlu-2xAdo	15	**41**	Lys^30^	30	C18 diacid	γGlu	97
**11**	Lys^13^	13	C18 diacid	γGlu-2xAdo	13	**42**	Lys^30^	30	C18 diacid	γGlu-4xAdo	75
**12**	Lys^14^	14	C18 diacid	γGlu-2xAdo	11	**43**	Lys^30^	30	C18 diacid	γGlu-6xAdo	78
**13**	Lys^15^	15	C18 diacid	γGlu-2xAdo	8.8	**44**	Lys^30^	30	C16	γGlu-2xAdo	0.5
**14**	Lys^16^	16	C18 diacid	γGlu-2xAdo	17	**45**	MeArg^35^	4	C18 diacid	γGlu-2xAdo	83
**15**	Lys^17^	17	C18 diacid	γGlu-2xAdo	39	**46**	Ala^4^, Lys^30^	30	C18 diacid	γGlu-2xAdo	84
**16**	Lys^18^	18	C18 diacid	γGlu-2xAdo	22	**47**	Arg^4^, Lys^30^	30	C18 diacid	γGlu-2xAdo	62
**17**	Lys^19^	19	C18 diacid	γGlu-2xAdo	29	**48**	Asp^18^, Lys^30^	30	C18 diacid	γGlu-2xAdo	104
**18**	Lys^20^	20	C18 diacid	γGlu-2xAdo	33	**49**	Ac, Ala^4^, Lys^30^	30	C18 diacid	γGlu-2xAdo	113
**19**	Lys^21^	21	C18 diacid	γGlu-2xAdo	34	**50**	Ac, Arg^4^, Lys^30^	30	C18 diacid	γGlu-2xAdo	120
**20**	Lys^22^	22	C18 diacid	γGlu-2xAdo	36	**51**	Ac, Ala^4^, Asp^18^, Lys^30^	30	C18 diacid	γGlu-2xAdo	114
**21**	Lys^23^	23	C18 diacid	γGlu-2xAdo	19	**52**	Arg^4^, Gln^18^, Lys^30^	30	C18 diacid	γGlu-2xAdo	79
**22**	Lys^24^	24	C18 diacid	γGlu-2xAdo	66	**53**	Lys^4^(TAMRA), Lys^7^	7	C18 diacid	γGlu-2xAdo	nd
**23**	Lys^25^	25	C18 diacid	γGlu-2xAdo	55	**54**	Lys^4^(TAMRA), Lys^10^	10	C18 diacid	γGlu-2xAdo	nd
**24**	Lys^26^	26	C18 diacid	γGlu-2xAdo	41	**55**	Lys^4^(TAMRA), Lys^25^	25	C18 diacid	γGlu-2xAdo	nd
**25**	Lys^27^	27	C18 diacid	γGlu-2xAdo	52	**56**	Lys^4^(TAMRA), Lys^30^	30	C18 diacid	γGlu-2xAdo	nd
**26**	Lys^28^	28	C18 diacid	γGlu-2xAdo	62	**57**	Lys^4^(TAMRA), Lys^33^	33	C18 diacid	γGlu-2xAdo	nd
**27**	Lys^29^	29	C18 diacid	γGlu-2xAdo	39	**58**	Lys^4^(TAMRA), Lys^30^	30	C14 diacid	γGlu-2xAdo	nd
**28**	Lys^30^	30	C18 diacid	γGlu-2xAdo	76	**59**	Lys^4^(TAMRA), Lys^30^	30	C16 diacid	γGlu-2xAdo	nd
**29**	Lys^31^	31	C18 diacid	γGlu-2xAdo	75	**60**	Lys^4^(TAMRA), Lys^30^	30	C20-diacid	γGlu-2xAdo	nd
**30**	Lys^32^	32	C18 diacid	γGlu-2xAdo	49	**61**	Lys^4^(TAMRA), Lys^30^	30	acetyl	γGlu-2xAdo	nd
**31**	Lys^33^	33	C18 diacid	γGlu-2xAdo	56						

*nd* not determined

## Results

### Y_2_R potency depends on the fatty diacid acylation position

The fatty acid acylated peptides are listed in Table 1 with the type of fatty acid and linker displayed in Fig. 1a, while The Y_1_R Y_2_R and Y_4_R potency of the lipidation scan is shown in Table 2. Not surprisingly, many of the analogues, notably those with a fatty diacid in the C-terminal part, were all displaying very low receptor potency on all receptor subtypes. The C-terminus of PYY_3-36_ binds deeply in the pocket of the Y_2_R (Fig. 1b) and subsequently derivatization with a bulky fatty diacid in the C-terminal region (25 to 32) lowers Y_2_R potency, in most cases dramatically (Table 2). We did not include analogues with a fatty diacid in positions 33 to 36, since it has already been established that any changes to these residues completely abolish receptor potency and binding to all NPY receptors^32^ due to significant steric hindrance imposed by, for example, a fatty diacid. The models by Kaiser et al. and B. Xu et al. also support this conclusion^4,33^. Both describe a very close interaction of the Y_2_R with residues in positions 32 to 36, thus not leaving any space for a larger fatty diacid handle. However, in position 30, normally occupied by Leu^30^ and indeed very close to the binding pocket (Fig. 1c), there seems to be an opportunity to add a fatty diacid with a modest reduction in the Y_2_R potency, as observed for analogue **28**, which displayed approximately a 8-fold reduction in the Y_2_R potency and affinity (Tables 2 and 3). In the context of human pancreatic polypeptide (PP) and the Y_4_R, position 30 has also previously been shown to tolerate lipidation with palmitic acid^34^, further substantiating that PYY and PP both share a common receptor binding mode in their C-terminal region.

**Table 2:** Summary of potency profiles of all PYY analogues lipidated from the N-terminal to position 32. In all analogues the fatty diacid (FA) moiety C18 diacid-γGlu-2xAdo was attached to the side chain of a lysine residue (analogues **2**-**30** and **52**) or to the N-terminus (peptide **1**). pEC_50_ is calculated as the mean of at least three independent experiments, and the 95% confidence interval (CI) is given as [lower; upper]. The mean EC_50_ in nM is calculated from the mean pEC50. For analogue **9** and **20** (data marked with *) two independent experiments were recorded.

No	FA	Y_2_ receptor		Y_1_ receptor		Y_4_ receptor	
	Position	EC50 (nM)	pEC50 [95% CI]	EC50 (nM)	pEC50 [95% CI]	EC50 (nM)	pEC50 [95% CI]
PYY_3-36_	none	0.60	9.2 [9.2:9.1]	7.9	8.1 [8.2:7.9]	320	6.5 [6.7;6.4]
**1**	Nα	10	8.0 [8.3;7.8]	25	7.6 [7.9;7.3]	250	6.6 [6.6;6.5]
**2**	4	25	7.6 [8.1;7.6]	200	6.7 [7.0;6.7]	630	6.2 [6.5;6.4]
**3**	5	25	7.6 [7.8;7.4]	100	7.0 [7.3;6.7]	400	6.4 [6.5;6.2]
**4**	6	7.9	8.1 [8.3;7.9]	50	7.3 [7.5;7.1]	320	6.5 [6.7;6.3]
**5**	7	2.0	8.7 [9.0;8.3]	63	7.2 [7.2;7.1]	250	6.6 [7.3;5.9]
**6**	8	40	7.4 [7.6;7.2]	250	6.6 [7.0;6.3]	790	6.1 [6.2;5.9]
**7**	9	13	7.9 [8.2;7.6]	200	6.7 [6.9;6.5]	630	6.2 [6.4;6.1]
**8**	10	1.3	8.9 [9.3;8.6]	50	7.3 [7.6;7.0]	500	6.3 [6.6;6.0]
**9**	11	5.0	8.3 [8.6;8.1]	50*	7.3*[-.-;-.-*]	> 1000	<6.0
**10**	12	16	7.8 [7.8;7.7]	200	6.7 [6.8;6.5]	500	6.3 [6.4;6.1]
**11**	13	32	7.5 [7.8;7.2]	320	6.5 [6.7;6.4]	500	6.3 [6.5;6.0]
**12**	14	4.0	8.4 [8.8;9.9]	63	7.2 [7.5;6.8]	1000	6.0 [6.2;5.9]
**13**	15	10	8.0 [8.3;7.8]	63	7.2 [7.3;7.1]	1000	6.0 [6.1;6.0]
**14**	16	20	7.7 [8.2;7.3]	160	6.8 [7.3;6.2]	> 1000	6.0
**15**	17	5.0	8.3 [8.9;7.6]	100	7.0 [7.5;6.5]	500	6.3 [7.1;5.5]
**16**	18	7.9	8.1 [8.3;7.9]	63	7.2 [7.5;7.0]	400	6.4 [6.6;6.1]
**17**	19	4.0	8.4 [8.8;8.0]	63	7.2 [7.4;7.0]	500	6.3 [7.1;5.6]
**18**	20	20	7.7 [8.0;7.4]	790	6.1 [6.2;6.0]	> 1000	<6.0
**19**	21	4.0	8.4 [8.7;8.0]	130	6.9 [7.2;6.7]	> 1000	<6.0
**20**	22	2.0*	8,7*[-.-;-.-*]	40	7.4 [7.4;7,3]	500*	6.3*[−.−; −.−*]
**21**	23	13	7.9 [8.4;7.5]	63	7.2 [7.4;7.0]	400	6.4 [6.5;6.3]
**22**	24	50	7.3 [7.6;7.0]	400	6.4 [6.6;6.3]	790	6.1 [6.4;5.8]
**23**	25	79	7.1 [8.0;6.1]	500	6.3 [6.9;5.7]	630	6.2 [6.6;5.7]
**24**	26	40	7.4 [7.5;7.3]	320	6.5 [6.5;6.4]	500	6.3 [6.6;6.0]
**25**	27	32	7.5 [8.0;6.9]	320	6.5 [6.8;6.2]	400	6.4 [6.6;6.3]
**26**	28	32	7.5 [7.7;7.3]	1000	6.0 [6.2;5.9]	>1000	<6.0
**27**	29	500	6.3 [6.8;5.8]	500	6.3 [7.0;5.6]	790	6.1 [6.5;5.8]
**28**	30	5.0	8.3 [8.5;8.2]	630	6.2 [6.4;5.9]	79	7.1 [7.3;6.9]
**29**	31	79	7.1 [7.2;7.1]	320	6.5 [6.6;6.4]	630	6.2 [6.4;6.1]
**30**	32	50	7.3 [7.4;7.2]	>1000	<6.0	>1000	<6.0
**52**	30	4.0	8.4 [8.6;8.3]	500	6.3 [6.5;6.0]	40	7.4 [7.4;7.3]

**Table 3:** *In vitro* Y_2_R SPA binding as a function of fatty diacid position and fatty diacid length in the absence (0% HSA) or presence of serum albumin (2% HSA). p*K*_i_ is calculated as the mean of at least three independent experiments, and the 95% confidence interval (CI) is given as [lower; upper]. The mean *K*_i_ in nM is calculated from the mean p*K*_i_.

No	Protractor		Y_2_R SPA (0% HSA)		Y_2_R SPA (2% HSA)		Fold change (Ki)
	Pos.	Type	*K*_i_ (nM)	p*K*_i_ [95% CI]	*K*_i_ (nM)	p*K*_i_ [95% CI]	+/− HSA
PYY_3-36_	none	none	0.40	9.4 [9.4;93]	1.0	9.0 [9.1;8.9]	2.5
**2**	4	C18diacid-γGlu-2xAdo	25	7.6 [7.8;7.4]	790	6.1 [6.2;6.0]	32
**5**	7	C18diacid-γGlu-2xAdo	1.0	9.0 [9.2;8.8]	630	6.2 [6.4;5.9]	630
**8**	10	C18diacid-γGlu-2xAdo	0.79	9.1 [9.2;9.1]	130	6.9 [7.1;6.7]	160
**9**	11	C18diacid-γGlu-2xAdo	5.0	8.3 [8.4;8.2]	160	6.8 [7.1;6.6]	32
**17**	19	C18diacid-γGlu-2xAdo	4.0	8.4 [8.5;8.3]	630	6.2 [6.3;6.0]	160
**20**	22	C18diacid-γGlu-2xAdo	1.6	8.8 [8.9;8.7]	400	6.4 [6.6;6.0]	250
**23**	25	C18diacid-γGlu-2xAdo	100	7.0 [7.1;6.8]	> 1000	6.0	Nd
**27**	29	C18diacid-γGlu-2xAdo	320	6.5 [6.7;6.3]	> 1000	6.0	Nd
**34**	30	C14diacid-γGlu-2xAdo	10	8.0 [8.1;7.8]	63	7.2 [7.4;7.1]	6.3
**35**	30	C16diacid-γGlu-2xAdo	7.9	8.1 [8.3;8.0]	79	7.1 [7.3;6.9]	10
**28**	30	C18diacid-γGlu-2xAdo	3.2	8.5 [8.7;8.4]	790	6.1 [6.1;6.0]	250
**37**	30	C20diacid-γGlu-2xAdo	1.3	8.9 [9.0;8.8]	250	6.6 [6.8;6.4]	190

The lipidation scan revealed that a fatty diacid is best tolerated in position 10 (analogue **8**), which showed reductions in Y_2_R potency and affinity by less than a factor of two (Tables 2 and 3) and was therefore comparable to native PYY_3-36_. This was followed by position seven (analogue **5**) which exhibited an approximate three-fold reduction in Y_2_R potency and affinity. Other lipidated analogues that display a modest decrease in Y_2_R potency have a fatty diacid in positions 11, 14, 19, 21 and 22, all of which are also exposed to the solvent. The hydrophobic core of the PP fold in PYY_1-36_ is composed of Pro^2^, Pro^5^ and Pro^8^ in the poly-proline sequence, which engage in hydrophobic interactions with Leu^17^, Tyr^20^, Leu^24^ and Tyr^27^ in the amphipathic helical segment ^3^. Notably, Pro^5^, Pro^8^, Tyr^20^ and Leu^24^ were all more sensitive to fatty diacid substitution, whereas Leu^17^ was less critical. Surprisingly, Ser^13^, Glu^15^ and Glu^16^ were also sensitive, though they are not part of the core, with Ser^13^ being part of the loop and the two glutamic acids residing in the solvent-exposed part of the amphipathic helix.

### Half-life as a function of position of fatty diacid protractor

**Figure 2:**
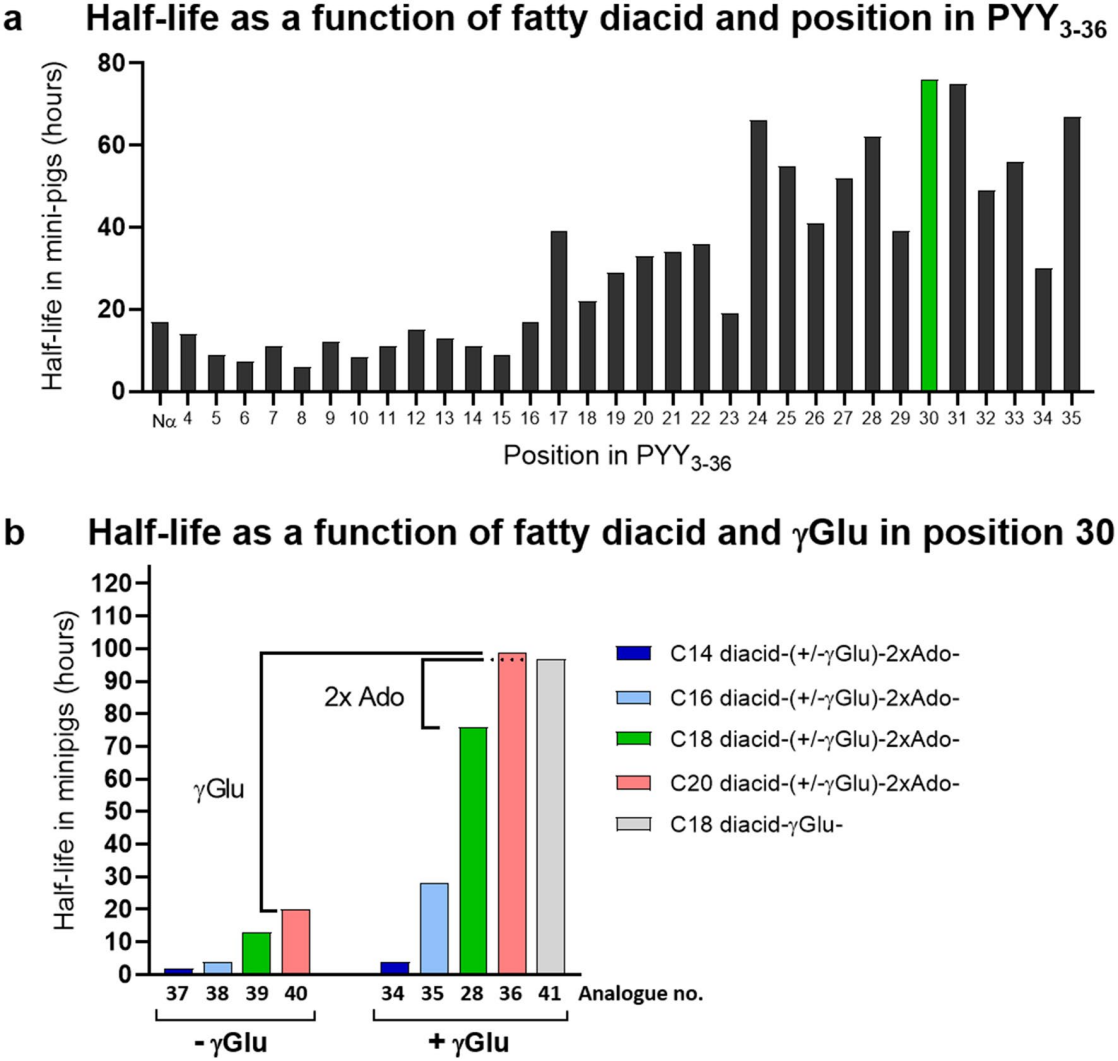
Plasma half-life in minipigs as a function of the position and type of fatty acid protractor. (**a**) Half-life in minipig using protractor C18diacid-γGlu-2xAdo as a function of position in PYY_3-36_. (**b**) Half-life as a function of fatty acid length and presence or absence of linker γGlu with all analogues having the protractor moieties placed in position 30. Half-lives are also listed in Table 1.

**Figure 3:**
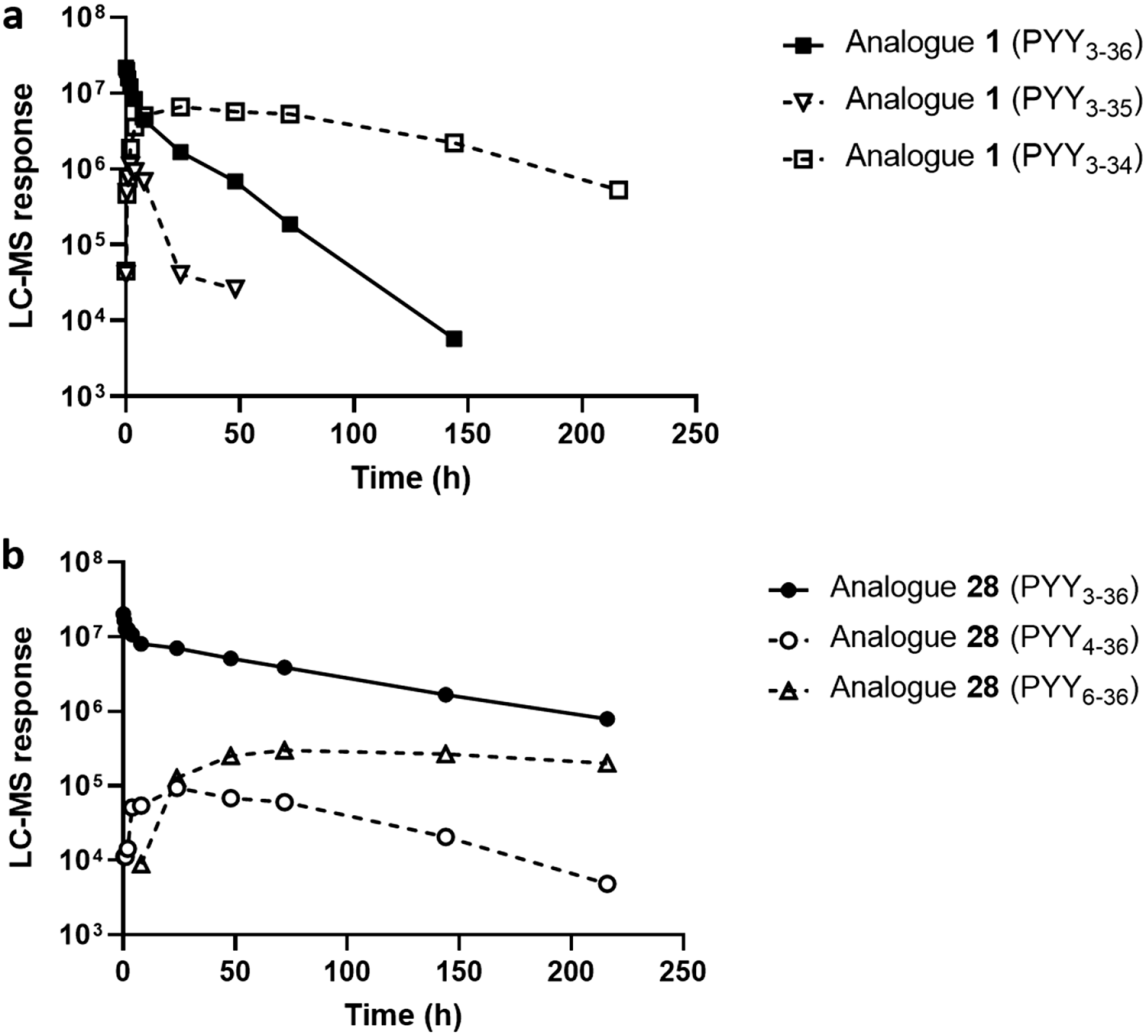
In vivo metabolism study in minipig. (**a**) Time profiles in minipig after dosing (i.v., 50 nmol/kg) of analogue **1** with fatty acid moiety on N-terminus and (**b**) analogue **28** with fatty acid moiety in position 30. Time profiles of identified degradation products are also shown (dotted lines). The LC-MS response was calculated from peak integration of the most abundant charge state.

As a protractor for the lipidation scan we chose C18 diacid-γGlu-2xAdo (Fig. 1a), proven to be adequate for once-weekly dosing in the GLP-1 analogue semaglutide. We observed a clear trend towards short half-lives around 10–20 hours when the fatty diacid moiety C18 diacid-γGlu-2xAdo was positioned towards the N-terminal end, whereas longer half-lives were observed when the fatty diacid was closer to the C-terminus, resulting in half-lives up to 60–78 hours (Table 1 and Fig. 2a). We hypothesized that attaching the fatty diacid towards the C-terminal end of PYY_3-36_ could have a shielding effect against the enzymatic degradation of the C-terminus to PYY_3-34_ and decided to investigate formation of metabolites *in vivo* in the minipig model. Indeed, we observed that analogue **1,** containing the fatty diacid moiety C18 diacid-γGlu-2xAdo at the N-terminus, rapidly metabolized to the corresponding PYY_3-34_ and PYY_3-35_ metabolites (Fig. 3a), whereas for analogue **28**, containing the fatty diacid protractor in position 30, we did not observe degradation of the C-terminus (Fig. 3b). However, in the MS spectrum of the plasma samples taken at 8 hours the N-terminal degradation products PYY_4-36_ and PYY_6-36_ were detected with analogue **28**, which, while small, were not detected in analogue **1**.

### Half-life as a function of type of lipidation in position 30

Lipidation with C18 diacid-γGlu-2xAdo in position 30 (analogue **28)** offered a sufficiently long half-life of 76 hours and could be considered as a good compromise between a long half-life, a decent Y_2_R affinity and a potency loss of only a factor 7–10 compared to PYY_3-36_. We therefore decided to explore the half-life in more detail as a function of the length of the fatty diacids as well as the role of the γGlu with the protractor fixed in position 30. This excluded the influence of the peptide backbone with respect to differences in proteolytic stability caused by variations in the position of the fatty diacid, thereby allowing us to focus the structure-activity relationship solely towards the fatty acid protractor with respect to half-life. The analogue with the shortest fatty acid C14 diacid-γGlu (analogue **34**) displayed a half-life of only 4 hours, which increased to 28, 76 and 98 hours for the C16 diacid-γGlu (analogue **35**), C18 diacid-γGlu (analogue **28**) and C20 diacid-γGlu (analogue **36**), respectively (Table 1 and Fig. 2b). We synthesized the same fatty diacid series without γGlu, and observed that this part of the protractor is of tremendous importance for achieving a long half-life. Within the analogue series **37**-**40** from C14 diacid to C20 diacid, but without γGlu, a significant reduction in half-life was observed, resulting in half-lives of only 2, 4, 13 and 20 hours, respectively (Fig. 2b). For the C20 diacid analogues the presence or absence of γGlu translates into a very large half-life difference of approximately 80 hours. The role of the spacer Ado was also addressed, but there was no difference with respect to half-life, whether 2xAdo (analogue **28**), 4xAdo (analogue **42**) or 6xAdo (analogue **43**) were inserted, since all three analogues displayed similar half-lives of 75–78 hours. Only when the spacer was absent and the C18 diacid-γGlu was directly attached to the side chain of the Lys^30^ residue (analogue **41)** did we observe an effect. Here the half-life increased to 97 hours (Table 1 and Fig. 2b), compared to half-lives of 75–78 hours with two or more Ado spacers. Most likely, this can be explained by a better shielding effect towards enzymatic cleavage by bringing the peptide backbone closer to albumin.

### Binding affinity of fatty diacid acylated analogues to albumin

For the different lengths of fatty diacids, plasma half-life appears to be driven by albumin binding affinity, whereas for the position of the fatty diacid, enzymatic cleavage of the PYY backbone seems to be the key determinant of the half-life, as opposed to differences in albumin affinity. In order to confirm these observations with a complementary assay, we measured the affinity of the PYY compounds to albumin in a direct binding assay (Table 4 and Supplementary Figure S4). For this purpose, the fluorescent dye 5(6)-Carboxytetramethylrhodamine (TAMRA) was coupled to the side chain of Lys^4^ in a series of PYY_3-36_ analogues, which allowed us to follow albumin binding by measuring the increase in fluorescence polarization (FP). To ensure reproducibility, fatty acid-depleted albumins was used, so the PYY peptide would not compete with already bound fatty acids. Under the assay conditions (50 nM peptide, *K*_D_ ~1 µM), not more than one PYY molecule is bound to one albumin molecule at any time, and the contribution of secondary binding sites on albumin is negligible.

**Table 4:** Binding affinity of TAMRA-labelled fatty diacid acylated PYY analogues to human (HSA) and porcine (PSA) serum albumin.

No.	Pos.	X-γGlu-2xAdo	HSA				PSA			
			*K*_d_ (µM) pH 7.4	p*K*_d_ [95% CI]	*K*_d_ (µM) pH 6.0	p*K*_d_ [95% CI]	*K*_d_ (µM) pH 7.4	p*K*_d_ [95% CI]	*K*_d_ (µM) pH 6.0	p*K*_d_[95% CI]
**53**	7	C18 diacid	1.5	5.8 [5.9;5.7]	nd	nd	1.7	5.8 [5.9;5.4]	nd	nd
**54**	10	C18 diacid	0.83	6.1 [6.2;6.0]	nd	nd	0.83	6.1 [6.2;6.0]	nd	nd
**55**	25	C18 diacid	2.0	5.7 [5.7;5.7]	nd	nd	2.2	5.7 [5.7;5.6]	nd	nd
**57**	33	C18 diacid	0.76	6.1 [6.2;6.0]	nd	nd	1.1	5.9 [6.1;5.8]	nd	nd
**58**	30	C14-diacid	44	4.4 [4.5;4.3]	23	4.6 [4.7;4.6]	48	4.3 [4.4;4.3]	34	4.5 [4.6;4.4]
**59**	30	C16-diacid	4.2	5.4 [5.5;5.3]	4.7	5.3 [5.5;5.2]	4.5	5.4 [5.4;5.3]	5.3	5.3 [5.4;5.2]
**56**	30	C18 diacid	0.58	6.2 [6.3;6.2]	0.93	6.0 [6.2;5.9]	0.60	6.2 [6.3;6.1]	0.89	6.1 [6.1;6.0]
**60**	30	C20-diacid	0.098	7.0 [7.1;6.9]	0.35	6.5 [6.7;6.2]	0.27	6.6 [6.7;6.5]	0.46	6.3 (6.6;6.1)
**61**	30	Acetyl	NB	–	–	–	NB	–	–	–

p*K*_d_ is calculated as the mean of three independent experiments, and the 95% confidence interval (CI) is given as [lower; upper]. The mean *K*_d_ in nM is calculated from the mean p*K*_d_.

*X* fatty diacid, *NB* no binding detected, *nd* not determined

Compounds with acylation in position 7 and 10 (analogues **53** and **54**) have a similar albumin binding affinity compared to compounds acylated in position 25 (analogue **55**), or to those in positions 30 and 33 (analogues **56** and **57**), even though their half-lives differ more than five-fold (Table 4). On average, the C18 diacid-γGlu-2xAdo side chain confers an albumin binding affinity of ~ 1 µM.

The relative length of the fatty diacid side chain had a significant effect on both human and porcine albumin binding affinity. We observed an increase by a factor of ten going from C14 diacid-γGlu (analogue **58**) to C16 diacid-γGlu (analogue **59**), an additional 8-fold increase from C16 diacid-γGlu to C18 diacid-γGlu (analogue **56**), and a 5-fold increase going from C18 to a C20 diacid-γGlu (analogue **60**), displaying a *K*_d_ of 0.1 µM (Table 4). This observation correlates well with the observed plasma half-lives of 4, 28, 76 and 99 hours, respectively.

We also wanted to explore whether the binding of our fatty acid acylated PYY analogues was pH dependent. By testing binding at pH 6.0, we could verify whether the fatty diacid acylated analogues were still capable of binding to HSA and PSA at lower pH. Indeed, for both the human and porcine albumins, we observed nearly the same binding affinities at pH 6.0 for the fatty diacid series as we do at pH 7.4 (Table 4 and Supplementary Figure S5).

### Half-life as a function of backbone modifications

We have previously reported that N-methyl-arginine in position 35 (MeArg^35^) stabilizes PYY_3-36_ against enzymatic degradation to PYY_3-34_ in an *in vitro* setting^35^. We therefore decided to include analogue **45** with the fatty diacid in position 4, and furthermore containing MeArg^35^. We then compared the half-life to the corresponding analogue without MeArg^35^ (analogue **2**), which by itself displayed a very short half-life of 14 hours. Indeed, a significant increase in half-life to 83 hours was obtained, thus confirming that, in an *in vivo* setting, protection against C-terminal cleavage is pivotal for obtaining longer half-lives. This proves that the primary reason for the observed shorter half-lives of the PYY analogues is the C-terminal cleavage of PYY_3-36_ to PYY_3-34_.

Since we observed small amounts of N-terminal degradation products for analogue **28**, which had a fatty diacid in position 30, we decided to explore additional modifications. The half-life of analogue **47** (Lys^4^ to Arg^4^ substitution) was unaffected; in fact, it was a little shorter (66 versus 76 hours) compared to that of native backbone. Similarly, a Lys^4^ to Ala^4^ substitution (**46**) had little impact on the half-life (84 hours). However, N-terminal acetylation combined with Lys^4^ to Arg^4^ substitution increased the half-life from 62 hours (**47**) to 120 hours (**50**), which supports the observation that an N-terminal modification could protect against additional proteolytic cleavage. Combining the Ala^4^ substitution with N-terminal acetylation, as in analogue **49,** resulted in a similarly long half-life of 113 hours. In PYY_3-36_, the residue Asn^18^ is known to deamidate. This process can be avoided by replacing Asn^18^ with Asp^18^, which exhibits good stability against isomerization at neutral pH and, in addition, also improves the formulation properties. We found that substituting Asn^18^ with Asp^18^ (analogue **48)** increased the half-life to 104 hours compared to the parent analogue **28**, a gain of approximately 28 hours. Previous studies of proteolysis of PYY_3-36_ suggested a potential cleavage site between Pro^14^-Glu^15^, among others^36^. It is reasonable to speculate that Asp^18^ might improve protection against degradation in this region. However, combining N-terminal acetylation with Ala^4^ and Asp^18^, as in analogue **51**, led to a half-life of 114 hours, which was similar to that of analogue **49** (113 hours) suggesting that the effects are not additive in this context.

### *In vivo* efficacy of a PYY analogue in combination with GLP-1

**Figure 4:**
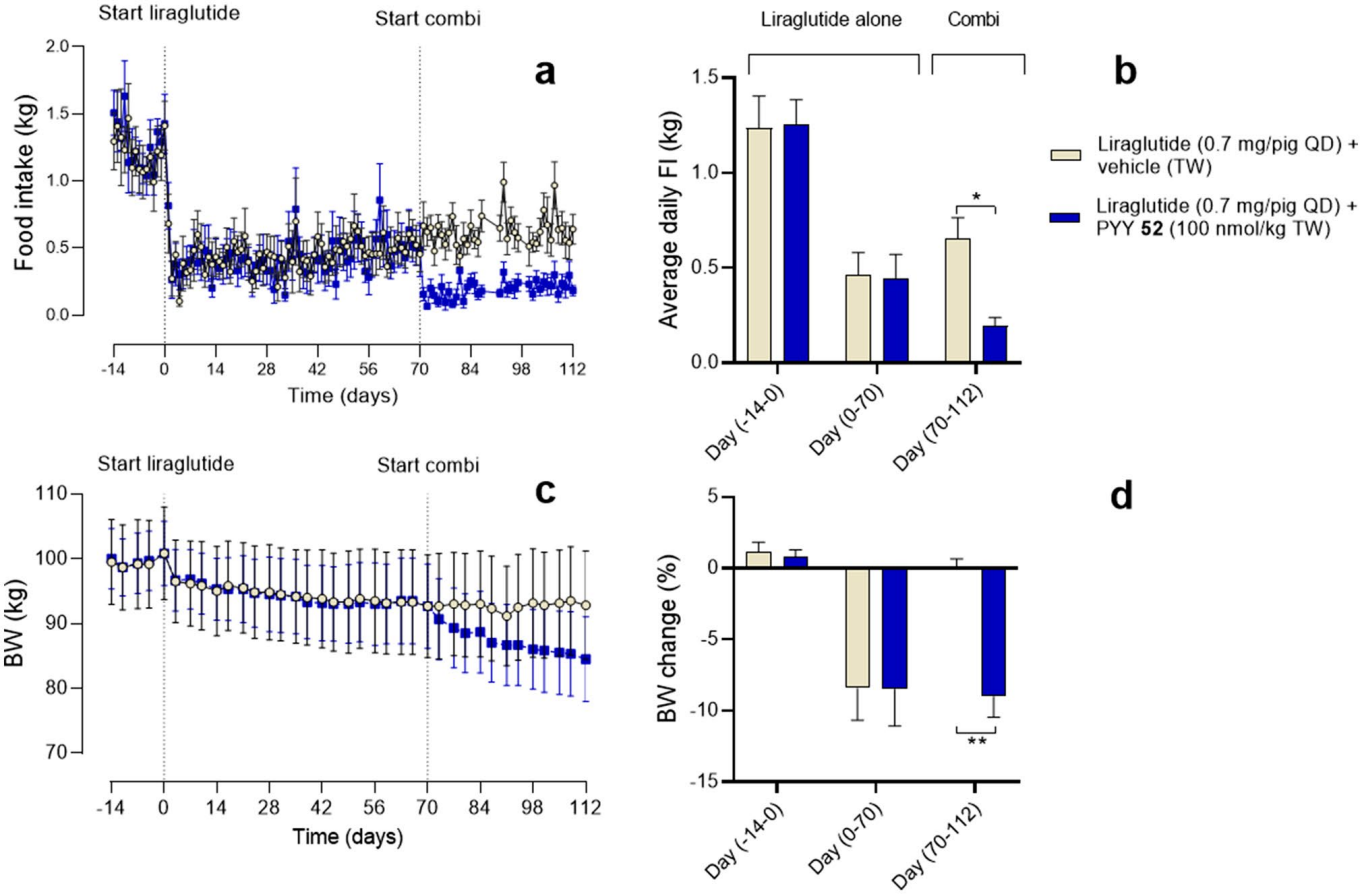
In vivo food intake study in Göttingen Minipigs (**a**) Daily food intake, (**b**) average daily food intake, (**c**) daily body weight and (**d**) average body weight change in baseline period (day -14 to day 0), liraglutide-alone period (day 70-112) and combination period (day 70-112) in obese OVX Göttingen Minipigs treated with liraglutide (0.7 mg/pig) for 16 weeks with add-on of vehicle (grey circle) or PYY analogue **52** (100 nmol/kg TW, black square) in the last 6 weeks. Statistical analysis involved two-way repeated measures ANOVA followed by Sidak´s multiple comparison test. Data presented as mean +/− SEM, n=6.

We designed an analogue **52** with Lys^4^ to Arg^4^ and Asn^18^ to Gln^18^ substitutions, with the former substitution allowing selective fatty diacid acylation only on Lys^30^ for ease of large-scale production, while the Gln^18^ was chosen to eliminate deamidation. Since the half-life of the compound in mini-pigs was 78 hours and the Y_2_R affinity and potency was only four to six-fold reduced compared to PYY_3-36_, this analogue was an attractive model compound for further *in vivo* evaluation in obese Göttingen Minipigs. Liraglutide alone reduced food intake by approximately 65% in both groups. When PYY was given in combination with liraglutide an additional, significant food intake reduction was observed and maintained until the termination of the experiment after 6 weeks of combination treatment. These reductions in food intake resulted in a BW loss of approximately 8.5% in the liraglutide-alone group, whereas the addition of analogue **52** resulted in an additional reduction of BW of approximately 9% from day 70-112, thus adding up to close to 20% of weight loss in total (Fig. 4).

## Discussion

Peptides are notoriously known to display a very short half-life in plasma, ranging from a few minutes to less than one hour, which in most cases results in a low or absent *in vivo* efficacy and therefore renders them unsuitable for therapeutic use. In particular, PYY_3-36_ has been shown to be degraded rapidly and cleared with a half-life of 5–10 minutes, necessitating the design of analogues with much longer half-lives to obtain *in vivo* efficacy. Lipidation of peptides is commonly used and considered to be a universal method to protract otherwise short-lived peptides. However, it is not a guarantee that by using this type of protractor moiety a long half-life is always obtained while also maintaining target receptor potency and proper formulation properties. In fact, as we show in this report, sufficient half-life is only obtained if the integrity of the peptide is also preserved. The fatty diacid acylation scan of PYY_3-36_ revealed that adding fatty diacids in close proximity to the C-terminus not only compromised the Y_2_R potency, but also showed that residues that are part of the core of the PP-fold are critical for maintaining good Y_2_R potency. However, we identified positions 7 (Ala^7^), 10 (Glu^10^) and 22 (Ser^22^) as positions where a fatty diacid protractor had only little impact on the Y_2_R potency (two to three-fold reduction compared to PYY_3-36_). This modest reduction in Y_2_R potency may partly be explained by the fact that the protractor is positioned farther away from the receptor, thus avoiding disruptive steric hindrance, but it is also likely rationalized by the preservation of the PP-fold, since both Ala^7^, Glu^10^ and Ser^22^ are exposed to the solvent (Fig. 1b).

We also measured the Y_2_R binding affinity in the presence of 2% albumin (HSA) and for all analogues the affinity was negatively impacted irrespectively of the position of the fatty diacid, when compared to the binding affinity measured in the absence of albumin. We observed 630 and 160-fold reduction in affinity for the analogues **5** and **7** which had a C18 diacid in position 7 and 10, respectively (Table 3). This was on the same level when the C18 diacid was present in position 30 displaying a 250-fold reduction in affinity. Since position 30 is closer to the receptor we would have expected a larger shift in Y_2_R affinity compared to the beforementioned analogues, where it is expected that the albumin may be farther away from the receptor. Only for the shorter C14 and C16 diacids, and thus displaying decreased HSA affinity, the shift in Y_2_R affinities was less affected which is the result of a larger unbound fraction of the PYY analogue. This shift in albumin binding does not hinder sufficient *in vivo* efficacy. The once-weekly and very potent GLP-1 analogue, semaglutide was in fact one of the analogues with the largest shift in the presence of 2% serum albumin^28^. When the fatty diacid was placed in position 30, we also observed a tendency of increased binding as a function of longer fatty acid length in the absence of serum albumin (Table 3). It can be speculated that the shorter fatty diacids may interact in a less favorable way with the negative charged residues that are present in the surroundings of the binding pocket in the Y_2_R.

The protractor C18 diacid-γGlu-2xAdo used in the fatty diacid acylation scan is the same albumin binder present in once-weekly GLP-1 analogue semaglutide, which displays a half-life of approximately 46 hours in the minipig model^28^ and approximately 168 hours in humans^37^. Surprisingly, attaching this protractor to PYY_3-36_ resulted in many analogues with much shorter half-lives in minipigs than reported for the once-weekly dosed semaglutide, even as short as only six hours. We observed a clear trend towards shorter half-lives when the protractor was present in the N-terminal part, whereas the half-lives increased above 50 hours when it was positioned towards the C-terminal end (Fig. 2a). By analyzing minipig plasma samples for metabolites, we concluded that PYY_3-36_ is rapidly cleaved to PYY_3-34_ while bound to albumin, and that by placing the fatty diacid moiety closer to the C-terminus, we could shield the peptide against enzymatic degradation. In addition, the fatty diacid moiety *per se* could also prohibit the enzymatic cleavage by imposing steric hindrance. Both shielding effects could explain the differences in half-lives, but the differences in albumin binding affinity could also play a role. In order to rule out that the major differences in observed half-lives were caused by differences in albumin affinity, we performed a FP assay to determine the affinity of analogues for both the human and porcine serum albumin. By comparing the affinities observed for analogues with fatty diacid in positions 7, 10, 25, 30 and 33, which represent a range of half-lives from 11 to 76 hours, we concluded that albumin affinity could not explain differences in half-lives, since all analogues bound to albumin with similar *K*_d_s within the range of 0.5 to 2 µM. The FP albumin binding assay was also used to determine the albumin affinities as a function of fatty diacid length, fixed in position 30. As expected, the fatty diacid length strongly affects the albumin affinity, with the shortest C14 diacid-γGlu displaying an affinity (*K*_d_) of only 40 µM for human albumin, which gradually increases as a function of fatty diacid length to 4.2, 0.5 and 0.1 µM for the C16 diacid-γGlu, C18 diacid-γGlu and C20 diacid-γGlu, respectively (Table 4). For porcine serum albumin we obtained very similar affinities for the same set of analogues, demonstrating a clear correlation between the albumin binding affinity as a function of fatty diacid length and the observed half-lives in minipigs. It also underlines that the minipig model is useful in order to rank and select analogues with an expected long half-life in humans, albeit allometric scaling using several species is needed for more a predictive half-life determination^38^. Both albumin and IgG display extraordinary long half-lives of approximately 19 days. They bind FcRn simultaneously in a pH dependent manner, with strong binding at pH 6.0 and much lower affinity at physiological pH^39^ which in turn ensures rescue of both albumin and IgG from degradation in the endothelial cells and hematopoietic cells. Since our analogues still bound almost equally well at pH 6.0 as at pH 7.4, we believe that the ultra-long half-lives we observe for some of analogues is also due to recycling by the FcRn while bound to albumin. This is a new observation and opens for the likelihood that the half-life of a peptide drug approaching the half-life of albumin itself may be achievable. This assumes that strong enough affinity to albumin is reached and the backbone is preserved from enzymatic degradation.

Not only the length of fatty diacid itself, but also the additional γGlu between fatty diacid and spacer are of importance. The influence of γGlu has previously been studied in the context of GLP-1 using the C16 fatty acid, palmitic acid, as the lipid moiety, and in this particular context, γGlu showed only a minor effect on the half-life^40^. Surprisingly, with the fatty diacids series used in this study, we observed huge differences in half-lives with or without γGlu. The C20 diacid series displayed a difference as large as 79 hours, whereas for the C18 diacid and C16 diacid series differences of 63 and 24 hours were observed, respectively (Fig. 2b). These differences representing a six to seven-fold change illustrate the importance of a negative charge that mimics the negative charge in the distal part of fatty acid and diacids. We also made three analogues in order to determine the effect of the spacer separating the fatty moiety from the peptide backbone. For analogue **41** with no Ado spacer, the half-life increased from 76 to 98 hours compared to analogue **28** which had two Ado spacers (Table 1 and Fig. 2b). This is likely due to a closer association of albumin to the peptide, thereby providing a better shielding effect against C-terminal degradation. Adding four or six Ado spacer units, as in analogues **42** and **43**, on the other hand did not affect the half-life (Table 1).

Since we observed that PYY_3-36_ is rapidly degraded to PYY_3-34_, despite being associated to albumin, we decided to design an analogue that was stabilized in the C-terminus in order to determine whether this strategy would also prolong the half-life *in vivo*. Previously, we have identified MeArg^35^ as a residue that could protect PYY_3-36_ against degradation to PYY_3-34_ in an *in vitro* setting. Indeed, the protracted analogue [MeArg^35^]PYY_3-36_ (analogue **45**) with C18 diacid-γGlu-2xAdo in position 4 displayed a much longer half-life (83 hours) than the comparator analogue **2** that had a half-life of only 14 hours. Although C-terminal degradation is dominant, other degradation pathways also occur *in vivo* while the peptide is associated to albumin. For the analogue **28**, that had a fatty diacid in position 30, we observed N-terminal degradation of PYY_3-36_ to PYY_4-36_ and PYY_6-36_, albeit to a smaller extent than the C-terminal degradation of PYY_3-36_ to PYY_3-34_. In contrast, we did not observe N-terminal cleavage in analogue **2**, with the fatty diacid in position 4. Again, this was likely due to a fatty diacid shielding effect and a relatively short half-life. After speculating whether the N-terminal stability could be improved, we designed analogues that should resist N-terminal degradation by e.g., N-terminal acetylation. Indeed, this dramatically extended the half-life, as illustrated by analogue [Ac, Arg^4^, Lys^30^]PYY_3-36_ (analogue **50**) with a half-life of 120 hours versus that of its comparator analogue [Arg^4^, Lys^30^]PYY_3-36_ (analogue **47**) which had a half-life of 66 hours. Replacing the Arg^4^ with Ala^4^ had a smaller positive effect of extending the half-life, as illustrated in [Ala^4^, Lys^30^]PYY_3-36_ (analogue **46**) which had a half-life of 84 hours. Again, we could increase this to 113 hours by using N-terminal acetylation, as in analogue [Ac, Ala^4^, Lys^30^]PYY_3-36_ (analogue **49**). Furthermore, we also identified Asp^18^ to increase the half-life from 76 hours (analogue **28**) to 114 hours, as in analogue [Asp^18^, Lys^30^]PYY_3-36_ (analogue **51**), again emphasizing the fact that several degradation pathways are in play for the metabolism of PYY_3-36_, although the degradation of PYY_3-36_ to PYY_3-34_ is dominant. Previous studies of proteolysis of PYY_3-36_ have suggested a potential cleavage site between Pro^14^- Glu^15^ among others^36^, and we hypothesize that Asp^18^ might improve protection against degradation in this region. The half-life of porcine serum albumin has been determined to approximately 8.2 days (197 hours)^41^. In general, we observe an upper limit of half-lives in the mini-pig model in the range of 120-140 hours, illustrating that we have likely reached the upper level in this model with respect to the ranking of these ultra-long acting analogues.

The combination of analogues of GLP-1 and PYY has been shown to act synergistically or additively in rodents^13^, in monkeys (s.c. every 3 days) and in humans (continuous infusion)^14,42,43^. We dosed obese minipigs with a protracted PYY analogue **52**, [Arg^4^, Gln^18^, Lys^30^]PYY_3-36_, which had a fatty C18 diacid in position 30, in combination with the once-daily GLP-1 analogue, liraglutide, to validate the concept. In the run-in period of 16 weeks with dosing of liraglutide alone for both groups, we observed a reduction in food intake of approximately 65%. Starting on day 70, group one was dosed with liraglutide alone, while the other group was dosed with a combination of liraglutide and analogue **52**. While group one receiving only liraglutide lost in total 8.5% in body weight after an additional 6 weeks of treatment, the group receiving the combination lost almost 20%. If the treatment period had been extended, potentially even greater losses may have been reached, thus confirming the great efficacy when combining GLP-1 and PYY for obesity treatment. Although we observe moderate potency on the Y_4_ receptor (40 nM) for analogue **52**, we do not believe that the observed additive effect in combination with liraglutide is also contributed by Y_4_ receptor agonism, since we have observed even greater weight losses with other PYY analogues that do not bind to the Y_4_ receptor (manuscript in preparation).

In conclusion, we have thoroughly investigated the effect of fatty diacid acylation of native PYY_3-36_ with respect to half-life, albumin binding and receptor potency. We have shown that position and fatty diacid length, in combination with stabilization of the peptide backbone, are essential for obtaining sufficient potency and much longer half-lives in the minipig model beyond what has been described for other fatty diacid protracted analogues, such as the once-weekly dosed semaglutide. Peptides as pharmaceuticals have become of increasing interest the last couple of decades within a broad area of diseases like diabetes and obesity, but also within gastrointestinal disorders, inflammation, and cancer. Notably, using display technologies peptides as well as small protein scaffolds are becoming popular in the discovery of *de novo* compounds that could disrupt protein-protein interactions (PPIs). We believe our observations provide valuable information with respect to the design of protracted peptides that would be of potential therapeutic value.

## Methods

Where relevant all methods were carried out in accordance with relevant guidelines and regulations. The *in vivo* procedures were approved by the Danish Animal Experiments Inspectorate and in accordance with ARRIVE guidelines.

### Peptide synthesis

The synthesis of individual peptides used for the *in vitro* assay and *in vivo* experiments was done using Prelude instruments (Protein Technologies) Peptides were purified by preparative HPLC, except peptides used for the PK experiments which were synthesized in plate format using Intavis MultipepRS equipment and used as crude peptides. For details about synthesis, cleavage and purification of the peptides see Supplemental Information.

### ACTOne functional potency assay

Assay principle: NPY Receptors are Gi-coupled seven trans-membrane receptors that signal through the cAMP-dependent pathway by inhibiting adenylate cyclase activity which results in a decrease of cAMP generation. The ACTOne assay is based on a modified calcium channel that has a selective binding site for cAMP, resulting in cellular calcium influx in presence of cAMP, detected by a calcium responsive dye. In order to measure decreased levels of cAMP, isoproterenol, a β1/β2-adrenoreceptor agonist, is added to activate adenylate cyclase and increase intracellular cAMP levels. Decreased cellular calcium concentrations, reflecting a decrease of cAMP due to YR activation, is detected as a decrease in fluorescence from the calcium sensitive dye. The human NPY receptors used in the assay are: Y_1_R UniProtKB: locus NPY1R_HUMAN, accession P25929, Y_2_R UniProtKB: locus NPY2R_HUMAN, accession P49146, Y_4_R UniProtKB: locus NPY4R_HUMAN, accession P50391.

Assay procedure: Human embryonic kidney (HEK) 293 cells stably expressing the cAMP sensitive calcium channel and one of the human Y_1_R, Y_2_R, and Y_4_R (Cat # CB 80300-244, CB 80300-245, CB 80300-270, Codex Bio-solution, Gaithersburg, MD, USA) were seeded into poly-lysine coated 384 well plates (Cat # 356663, BD Biosciences, Franklin Lakes, NJ, USA) at a density of 14000 cells/well in a volume of 25 µl in DMEM (Lonza) containing fetal bovine serum (Cat # 16140-07110, Gibco), 1% Penicillin-Streptomycin (Lonza), 250 µg/ml G418 (Cat # 10131027, Gibco), 1 µg/ml Puromycin (Sigma-Aldrich) and 0.1 mM albumin binding eicosanedioic acid-Glu-Glu (NNC0069-0010 Novo Nordisk, Måløv, Denmark, molecular weight 600.76 Da) in order to prevent albumin interaction. The cells were incubated for 2 days at 37 °C in a humidified milieu in 5% CO_2_. On the day of the assay, 25 µl calcium dye buffer containing: 1 vial Calcium 5 dye (Cat # 5000625, Molecular Devices, Sunnyvale, CA, USA) dissolved in 100 ml HBSS (Cat #14025092, Gibco) containing 20 mM HEPES, 1.5 mM probenecid (Cat #P8761, Sigma-Aldrich), 250 µM PDE-inhibitor 4-(3-Butoxy-4-methoxybenzyl)imidazolidin-2-one (Cat # B8279, Sigma-Aldrich) and 0.129 mM CaCl_2_ pH 7.40 was added to each well (total volume 50 µl/well). Cells were incubated for 1 hour at 37 °C followed by 1 hour at room temperature protected from light. Cell plates were then placed individually in a FLIPR Tetra System (Molecular Devices) where the liquid handling system added 1 μl PYY analogue and 1 μl isoproterenol (0.05 µM final concentration, Cat # I6504, Sigma-Aldrich) simultaneously, directly followed by fluorescence signal measurements (Ex540/Em590) with 30 seconds intervals. All measurements were performed in duplicates and EC_50_ values were calculated by nonlinear regression analysis of sigmoidal dose response curves at the time point 360 seconds, using GraphPad Prism (Graph Pad software 9.0.1, www.graphpad.com, La Jolla, CA, USA).

Serial dilution: Stock solutions of PYY analogues (200 µM) in 80% dimethyl sulfoxide (DMSO), 19% H_2_O and 1% acetic acid were serially diluted in analogue buffer containing Hank's balanced salt solution (HBSS) (Cat # 14025092, Gibco), 20mM Hepes (Cat # H3375, Sigma-Aldrich), 0.1% ovalbumin (Cat # A5503, Sigma-Aldrich), 0.005% Tween 20 and 30% DMSO. Final assay concentrations ranged from 0.03 nM to 30 nM for Y_2_R assays and 1 nM to 1000 nM for Y_1_R and Y_4_R assays. Reference analogues PYY_3-36_ and PP_1-36_ were serially diluted ranging from 0.03 nM to 30 nM in all assays.

### Y_2_R Scintillation Proximity Assay (SPA) binding assay

Human Y_2_R cell culture: CHO cells stably expressing the human Y_2_R (Cat # CG1274, Multispan, Hayward, CA, USA) were cultured in DMEM F-12 (Cat # 31331-028, Gibco) with 10% heat inactivated fetal bovine serum (Gibco), 1% Penicillin-Streptomycin (P/S) (Lonza), 150 µg/ml Hygromycin B (Merck KGaA) and 10 µg/ml Puromycin (Cat # P8833, Sigma-Aldrich).

Y_2_R membrane preparation: Cells were cultured in 500 cm^2^ Nunclon dishes (Cat # 166508, Thermo Fisher Scientific, Waltham, MA, USA) and detached mechanically by scraping. Plates were washed in ice cold DPBS (Cat # 17-512F, Lonza) and cells were transferred to tubes and centrifuged for 5 min at 1000 g at 4ºC in a Heraeus Multifuge 3s (Thermo Fisher Scientific). Pellets were resuspended in ice cold Y_2_R homogenization buffer (20 mM HEPES, 5 mM MgCl_2_, 1 mg/ml Bacitracin, pH 7.1) and homogenized for 30 seconds using an Ultra-Turrax T25 homogenizer (IKA, Staufen, Germany) at medium speed. The homogenate was centrifuged at 35000 g (18000 rpm) using a Sorvall RC6+ ultracentrifuge (Thermo Fisher Scientific) with a Fiberlite F21-8x50 rotor for 15 minutes at 4°C and the supernatant was discarded. Homogenization of the pellet was repeated a total of three times in fresh Y_2_ homogenization buffer. The final pellet was resuspended in Y_2_R homogenization buffer and protein concentration was determined and adjusted to 1 mg/ml with Y_2_R homogenization buffer. Protein concentration was determined by Bio-Rad protein assay kit II (Cat # 500-0002, Hercules, CA, USA).

Wheat germ agglutinin SPA beads (Cat # RPNQ 0001, PerkinElmer, Waltham, MA, USA) were reconstituted in Y_2_ SPA buffer (50 mM HEPES, 1 mM CaCl_2_, 5 mM MgCl_2_, 0.02% Tween 20, 0.25% ovalbumin pH 7.4) and mixed with membrane preparation to give a final concentration of 0.5 mg SPA beads and 3 µg/well total protein Serially diluted PYY analogue in Y_2_R SPA buffer was added followed by 50.000 cpm per well of human [^125^I]-Peptide YY_1-36_ (Chemistry and Isotope Lab, Novo Nordisk A/S) corresponding to a concentration of 50 pM of radio ligand. Protein concentration were adjusted to 1 mg/ml with Y_1_ homogenization buffer B and transferred to cryotubes and stored at − 80 °C.

Scintillation Proximity Assay (SPA): Y_2_R receptor binding assays were performed in 96-well Optiplates (Cat # 6005290, PerkinElmer) in a total volume of 200 µl per well. Stock solutions of PYY analogues (2000 µM) in 80% dimethyl sulfoxide (DMSO), 19% H_2_O and 1% acetic acid were serially diluted in Y_2_ SPA buffer.

Displacement of radio ligand was measured as reduction in light emission from SPA beads and pKi (human) or pIC_50_ (mouse) values were calculated by nonlinear regression analysis of sigmoidal dose-response curves in GraphPad Prism (Graph Pad software 9.0.1, www.graphpad.com). All dose response measurements were performed in technical duplicates and data presented represents a minimum of 3 independently performed assays.

Cheng-Prusoff equation was used for p*K*_i_ calculations

$$K_{{\text{i}}} = {\text{IC}}_{{{5}0}} /({1} + \left( {\left[ {{\text{Radioligand}}} \right]/K_{{\text{d}}} } \right)$$

Saturation binding: *K*_d_ is the equilibrium binding constant which corresponds to the radio ligand concentration needed to achieve a half-maximum binding at equilibrium. Radio ligand human [^125^I]-Peptide YY_1-36_ (Cat # NEX3410, PerkinElmer) was used for Y_2_R saturation binding experiments. In order to calculate specific binding, saturation binding was performed with increasing concentrations of radio ligand for total binding and for non-specific binding in the presence of 1 µM cold ligand. Cold ligand for Y_2_R was human PYY_3-36_. Calculation of *K*_d_ was performed where nonspecific binding was subtracted from total binding to determine the specific binding and fitted in one site binding (hyperbola) equation. Three independent saturation binding experiments were performed for each NPY receptor type and *K*_d_ values for each experiment were converted to p*K*_d_ (–log*K*_d_) followed by calculation of the mean p*K*_d_ and thereafter converted back (10^-mean p*K*d^) to mean *K*_d_. Y_2_R *K*_d_ = 275 pM.

### Albumin binding affinity of fluorescently labelled peptides

The albumin binding affinity of 5(6)-Carboxytetramethylrhodamine (TAMRA)-labelled, acylated PYY compounds was measured in a fluorescence polarization assay. A 12-point dilution series of human (HSA; Cat # A3782, Sigma) or porcine serum albumin (PSA; Cat # A4414, Sigma) was titrated to a constant peptide concentration of 50 nM in 10 mM sodium phosphate, 140 mM NaCl, pH 7.4 or 20 mM MES, 150 mM NaCl, pH 6.0. Albumin compounds were purchased free from fatty acids (HSA); or, fatty acids were removed by treatment with activated charcoal (PSA)^44^. In this manner, all binding sites in albumin are free from fatty acids, and the assay determines peptide binding to the albumin site with the highest affinity. Samples were incubated for 2 h at room temperature and fluorescence polarization was measured in duplicate in a 384-well plate (Cat # 784900, Greiner) on a microplate reader (Spark, Tecan), using 535/25 nm and 595/35 nm excitation and emission filters, respectively (a second measurement after 20 h confirmed that equilibrium was reached in the 2 h measurement). The binding curve was fitted with a 1:1 binding isotherm in GraphPad Prism (Graph Pad software 9.0.1, www.graphpad.com), using the equation $S = a\frac{x}{{K_{{\text{D}}} + x}} + b$, with *S* the measured fluorescence polarization, *b* the fluorescence polarization signal of the unbound peptide, *a* the amplitude of the signal change, *x* the albumin concentration and *K*_D_ the binding affinity. This treatment is warranted as the assay operates under pseudo-first order conditions (peptide concentration << *K*_D_), where the free concentration of albumin can be equated to the total concentration of albumin. A non-acylated PYY analogue having an acetyl in place for the fatty diacid (analogue **61**) was included as control and found not to exhibit any binding to albumin (see Supplementary Information).

### In vivo pharmacokinetic (PK) evaluation in Göttingen Minipigs

#### Animals and housing

Male Göttingen Minipigs with a body weight (BW) ranging from 10 to 25 kg (Ellegaard Minipigs, Dalmose, Denmark) were used for PK evaluations since this species has been shown to be a valid model for prediction of human PK^45^. The animals were either group-housed or single-housed depending on the presence of permanent central-venous catheters in the animal unit at Novo Nordisk A/S or BioAdvice A/S. They were housed in a 12 h light cycle and were allowed minimum 2 weeks of acclimatization before study start. They had wood shavings and/or straw as bedding material and were fed standard minipig chow with free access to water. In some animals, two permanent central-venous catheters were surgically implanted in the caudal caval vein according to previously described principles^46^. The number of animals used for these studies was based on previous experience with PK experiments performed in Göttingen Minipigs.

#### Administration of peptide formulations

The animals were fasted overnight prior to dosing but had ad libitum access to water. Each animal received an IV injection of 2–6 analogues in one formulation (3–4 animals per formulation, 15 nmol/kg of each analogue), given either through a venflon placed in the ear vein in briefly restrained pigs or through one of the IV catheters, which was flushed with minimum 10 ml of sterile saline post administration. The test substances were formulated in either 10 mM NaHPO_4_, 150 mM NaCl, 0.01% Tween 20, pH 4.0 or 50 mM Na_2_HPO_4_, 145 mM NaCl, 0.05% Tween 80, pH 7.4 and were dosed in a volume of 0.1–0.15 ml/kg.

#### Blood sampling

Blood samples (0.8 ml) were taken either from the jugular vein using vacutainer or from the IV catheter not used for dosing according to one of the following schedules: Predose, and 5, 15, 30, 45 min, 1 h, 1.5 h, 2 h, 3 h, 4 h, 6 h, 8 h, 10 h, 24 h, 48 h, 72 h,96 h, 120 h, 168 h, 192 h, 216 h, 240 h, 264 h and 288 h post dosing; or Predose, and 5, 30 min, 1 h, 2 h, 4 h, 7 h, 11 h, 24 h, 48 h, 72 h,96 h, 120 h, 168 h, 216 h, 264 h post dosing. Blood was collected in tubes containing EDTA buffer (8 mM) and 50 µl stabilization buffer (3.097 g K_3_EDTA dissolved in 50 ml aprotinin Trasylol^®^, 10,000 KIE/ml) and added 0.5 ml 20 mM valine-pyrrolidide (prepared at Novo Nordisk A/S); pH regulated to 7.4). Samples were kept on wet ice until centrifugation (10 min, 4 °C, 1500- 2000g). Afterwards, plasma was transferred to Micronic tubes and kept at - 20 °C until analysis. The plasma samples were analysed by LC/MS as described below. Plasma concentration-time profiles were analysed by a non-compartmental pharmacokinetics analysis using Phoenix WinNonlin v. 5.0 (Pharsight Inc., Mountain View, CA, USA). Calculations were performed using individual concentration-time values from each animal, and minimum 3 data points were used for estimation of terminal half-life.

#### Subchronic in vivo efficacy study in obese Göttingen Minipigs

Obese, female ovariectomised Göttingen Minipigs (Göttingen Minipigs, Dalmose, Denmark) weighing 101 ±14 kg (range:78–130 kg) were used to evaluate the weight lowering effect of treatment with PYY analogue 52 in combination with the GLP-1 analogue liraglutide. The obese minipig model, with an overweight of more than 100%, is suitable for this type of study due to a large treatment window compared to rodents, which is essential for evaluation of the full weight lowering potential of a combination treatment. The pigs were single-housed in pens of at least 4 m^2^ floor area, with wood shavings and straw as bedding material and a 12 h light cycle. The pigs were fed ad libitum on Altromin 9023 (Brogaarden, Denmark) and had free access to water. After a baseline period of 2 weeks, all pigs were treated for a period of 16 weeks with liraglutide (190 nmol/pig given subcutaneously (SC) once daily), and during the last 6 weeks of this period, add-on treatment with either the PYY analogue **52** in a dose of 100 nmol/kg or vehicle was given twice weekly. The study was approved by the Danish Animal Experiments Inspectorate. The number of animals used was based on previous experience with appetite-regulating compounds in this model.

#### Quantitative assay for plasma samples

The PYY analogues from cassette dosing studies were quantified in plasma samples by LC/MS/MS. The selectivity of the method allowed multiple analogues to be quantified in the same plasma sample. Calibration curves were constructed by spiking PYY analogues to blank plasma and method performance was accepted based on standard curves and quality control samples in duplicate at three concentration levels. The typical dynamic range of the assay was 1 - 2,000 nmol/l. The sample preparation was as follows: 40 µl of EDTA plasma was added to 160 µl of 50% methanol containing 1% formic acid, then vortexed and centrifuged at 14300 rpm (16457 g) at 4 ºC for 20 minutes. The supernatant was transferred to a 96 well plate and the injection volume was 25 µl. For sample clean-up procedure, a TurboFlow Cyclone column (0.5 × 50 mm) from Thermo Scientific, Franklin, MA, USA, was used and the chromatographic separation was performed on an Onyx C18 column (2.0 × 50 mm) from Phenomenex (Torrance, CA, USA). Mobile phases were composed of combinations of methanol, acetonitrile, Milli-Q water and formic acid. Selective detection of PYY analogues by multiple reaction monitoring was performed on a API3000 mass spectrometer from Sciex (ON, Canada) that was operated in positive ionization mode.

#### In vivo metabolism studies in Göttingen Minipigs

In order to identify degradation products, two minipigs were dosed i.v. with 50 nmol/kg PYY analogues and plasma samples taken according to the procedures described for *in vivo* pharmacokinetic studies. Plasma samples were analyzed by LC/MS on an LTQ-Orbitrap (ThermoFisher Scientifc, Bremen). Analysis and identification were performed according to procedures previously described^9^. Briefly, aliquots of plasma from two minipigs were pooled for each time point. 30µl of plasma was precipitated with 90µl of ethanol and after centrifugation, the supernatant was diluted with one volume of water prior to LC/MS analysis. The mass spectrometer was equipped with an electrospray interface, which was operated in positive ionization mode. Analysis was conducted in a full scan mode from m/z 300–1800. HPLC was performed on a Jupiter Proteo column (4µ) 90A (50 × 2.0 mm ID). Mobile phases consisted of A. 0.1% formic acid and B. 0.1% formic acid in acetonitrile. A gradient was run from 5% B to 60% B over 10 minutes at a flow rate of 0.3 ml/min.

### Materials

All Fmoc-amino acids and Oxyma Pure were purchased from Protein Technologies (now Gyros Technologies) or Novabiochem, Merck. Fmoc-L-Glu-Otbu (γGlu), Fmoc-L-Lys(Mtt)-OH and Fmoc-L-Lys(ivDde)-OH were obtained from Iris biotech (Germany). Dimethylformamide (DMF) was from Solvias (Switzerland). Fmoc-8-amino-3,6-dioxaoctanoic acid (Fmoc-Ado-OH) was purchased from Flamma Group, Italy. 20-(tert-Butoxy)-20-oxooctadecanoic acid (C20-diacid), 18-(tert-Butoxy)-18-oxooctadecanoic acid (C18-diacid), (16-(tert-Butoxy)-16-oxooctadecanoic acid (C16-diacid) and 14-(tert-Butoxy)-14-oxooctadecanoic acid (C14-diacid) were purchased from Solvias. Fmoc-PAL-resin, trifluoroacetic acid, diethylether, piperidine, acetonitril, hydrazine and triisopropylsilan were from Merck. 5(6)-TAMRA (AS-81120) was purchased from Anaspec (USA). All buffers and salts for *in vitro* were purchased from Sigma Aldrich. ActOne assay: Human embryonic kidney (HEK) 293 cells stably expressing the cAMP sensitive calcium channel and one of the human Y1R, Y2R, and Y4R (Cat # CB 80300-244, CB 80300-245, CB 80300-270, Codex Bio-solution, Gaithersburg, MD, USA). SPA assay: CHO cells stably expressing the human Y_2_R (Cat # CG1274, Multispan, Hayward, CA, USA).

## Supplementary Information

Supplementary Information.

## Data Availability

The data that support the findings of this study are available on reasonable request from the corresponding author.

## Abbreviations

cAMP: cyclic adenosine monophosphate
DPP-IV: dipeptyl peptidase 4
Fmoc: fluorenylmethyloxycarbonyl
GLP-1: glucagon-like peptide-1
HBSS: Hank’s balanced salt solution
HEK: human embryonic kidney
HEPES: 4-(2-hydroxyethyl)-1-piperazineethanesulfonic acid
HSA: human serum albumin
NPY: neuropeptide Y
PDE: phosphodiesterase
PP: pancreatic polypeptide
PSA: porcine serum albumin
PYY: peptide YY
SPA: scintillation proximity assay
SPPS: solid-phase peptide synthesis
Y_1_R, Y_2_R, Y_4_R: NPYR subtypes 1, 2, and 4, respectively
