# Supplementary material for: The effect of fatty diacid acylation of human PYY3-36 on Y2 receptor potency and half-life in minipigs
Source: Sci Rep. 2021 Oct 27;11:21179. doi: 10.1038/s41598-021-00654-3 (PMC8551270; doi:10.1038/s41598-021-00654-3)
Supplement: Supplementary file 1 — Supplementary Information. [file 41598_2021_654_MOESM1_ESM.docx]

**Supporting Information**

**The effect of fatty diacid acylation of human PYY_3-36_ on Y_2_ receptor potency and half-life in minipigs**

Søren Østergaard*^1^, Johan F. Paulsson^1^, Jacob Kofoed^1^, Franziska Zosel^1^, Jørgen Olsen^1^, Claus Bekker Jeppesen^1^, Jane Spetzler^1^, Lars Ynddal^2^, Luise Gram Schleiss^1^, Berit Østergaard Christoffersen^1^, Kirsten Raun^1^, Ulrich Sensfuss^3^, Flemming Seier Nielsen^1^, Rasmus Jørgensen^4^, and Birgitte S. Wulff^1^

*Corresponding author

^1^Novo Nordisk A/S, Novo Nordisk Park, 2760 Maaloev, Denmark

^2^Gubra Aps, Hørsholm Kongevej 11B, 2970 Hørsholm, Denmark

^3^STipe Therapeutics, Copenhagen, Denmark

^4^CitoKi Pharma, Værløse, Denmark

**Contents of supporting information**

**Materials and methods peptide synthesis** p. S1-S3
**Table S1** with purity found mass p. S3-S5
**Table S2**. UPLC gradients specified p. S6
**ADMET** p. S6-S8
**FP assay** p. S9-S10

**1. Materials and methods peptide synthesis**

All solvents and chemicals were used without any further purification. Materials. N-methylpyrrolidinone (NMP) was purchased from Biosolve. Diisopropylcarbodiimide and collidine was purchased from Sigma. The following L-amino acids were used: Fmoc-Ala, Fmoc-Arg(pbf)-OH, Fmoc-Asn(Trt)-OH, Fmoc-Asp(Otbu)-OH, Fmoc-Gln(Trt)-OH, Fmoc-Glu(Otbu)-OH, Fmoc-Glu-Otbu, Fmoc-Gly-OH, Fmoc-His(Trt)-OH, Fmoc-Ile-OH, Boc-Ile-OH, Fmoc-Leu-OH, Fmoc-Val-OH, Fmoc-Lys(boc)-OH, Fmoc-Lys(Mtt)-OH, Fmoc-Ser(Otbu)-OH, Fmoc-Thr(Otbu)-OH, Fmoc-Tyr(Otbu)-OH, FmocTrp(Boc)-OH, Fmoc-Phe-OH, and they were purchased from either Protein Technologies, Novabiochem Merck or IRIS. Rink amid PS resin, PAL resin PS resin were purchased from Novabiochem. Fmoc-8-amino-3,6-dioxaoctanoic acid (Fmoc-Ado-OH) was purchased from Flamma Group, Italy. 20-(tert-Butoxy)-20-oxooctadecanoic acid (C20-diacid) ,18-(tert-Butoxy)-18-oxooctadecanoic acid (C18-diacid), (16-(tert-Butoxy)-16-oxooctadecanoic acid (C16-diacid) and 14-(tert-Butoxy)-14-oxooctadecanoic acid (C14-diacid) was purchased from Solvias.

**Methods peptide synthesis, purification and analysis**

Peptides for *in vitro* experiments were synthesized using Prelude or Prelude X with heating. Instruments were equipped with 0,3M solutions of Fmoc-amino acids that were solubilized with either 0,3M HOBt or 0,3M Oxyma Pure in NMP or DMF. In situ activation was done by adding 1/10 volume (relative to amino acid volume) of 3M DIC in NMP and 1/10 volume 3M collidine in NMP. A final excess of 6-8-fold activated amino acid relative to resin substitution was typically used and coupling time was 60-90 min without heating whereas 10-15 min was used with heating at 60 ^°^C. For some of the peptides Fmoc-Arg(pbf)-OH was coupled using double couplings. Removal of the Fmoc-group was done by adding 2x resin volume 25% piperidine in NMP (4 + 4 min.) but only 1+2 min with heating. The N-terminal Ile was coupled as Boc-Ile-OH using conditions as described for the Fmoc-amino acids. The Mtt protecting group on lysine was removed with 4x washing with 3% triisopropylsilane (TIPS) in 75% HFIP in 25% DCM and with a total of 30 min of cleavage time. The fatty acid albumin binder C18diacid-γGlu-2xAdo and other protractors was synthesized by stepwise assembly using the procedures as described for backbone synthesis with the exception that coupling times were prolonged to 2-6 hours. Fatty diacids were used as mono-tert-butyl protected fatty acids and purchased from Solvias. After synthesis the peptidyl-resins were washed with DCM and dried. The peptides were purified by preparative HPLC using a linear gradient: 20-50% ACN with 0.1% TFA (buffer B) (alternatively 10-30% buffer B) over 40 min on a SymmetryPrep C18 (19 × 300 mm, 7 µm column) (Waters Corporation, Milford, USA) eluting at 20 ml/min. Analysis of purity of the peptides was performed using Waters Acquity UPLC system, with Waters Acquity TUV detector 214 nm and 254 nm. For column details and gradients used see Table 1+2. For some peptides the molecular weights were determined using matrix-assisted laser desorption and ionization time-of-flight mass spectroscopy, recorded on a Microflex or Autoflex (Bruker Daltonics, Bremen, Germany). A matrix of α-cyano-4-hydroxy cinnamic acid was used. Alternatively, characterization was performed by UPLC–MS on a setup consisting of a Waters Acquity UPLC system connected to an LCT Premier XE mass spectrometer from Micromass, or by HPLC–MS on an Agilent 1200 series HPLC connected to an Agilent 6230 time-of-flight (TOF) system using solvent A 0.1% formic acid in H_2_O and solvent B 0.1% FA in ACN. All the purified analogues were dissolved in 80% DMSO with 20% H_2_O as stock solutions. All peptide stocks for in vitro assays were quantified to a final concentration close to 200 µM by using a chemiluminescent nitrogen detector (Thermo Scientific Vanquish) or charged aerosol detector (Thermo Scientific Ultimate 3000) before doing in vitro assay. The measured concentration was used in data analysis.

**Peptides for PK experiments in mini-pigs**Parallel synthesis of crude acylated peptides for in vivo PK was done in 96 filter plate format using Intavis Multipep RS equipment. Each peptide was synthesized in four wells that later were combined. Solid phase of backbone was done using reagents as described above with triple couplings for 15 min, 60 min and 90 min. Approximately 10 mg of Fmoc-Rink amide Tentagel resin (Rapp Polyemre , Germany was placed in each well. Coupling volume for each well was 120 ul (80 ul 0,3M Fmoc-AA-OH in 0.3M oxyma pure in NMP with 40 ul 1M DIC in NMP). Deprotection with 25% piperidine in NMP was done by adding 150 ul to each well twice for 2 min and 8 min, respectively. After synthesis the resins were washed with ethanol and dried. The resin filter plate was placed in top of a 2 ml deep well plate and TFA deprotection (4% trisisopropylsilane, 3% H_2_O in TFA) was added three times 250 ul of TFA. Within the first 5 min 3 x 250 ul was added then allowed to stand for 30-60 min. Then another 250 ul TFA was added and allowed to stand for 60 -90 min. A final 250 ul TFA was added and the TFA in the deepwell plate was reduced in volume to approximately 150 µl pr well prior to the addition of diethyl ether (850 µl pr well) The precipitated peptides were then transferred to Solvinert filter plate (Millipore) by a multichannel pipette and washed with diethyl ether (4x 500 µl pr well). The peptides were dried and dissolved in 50% aqueous acetic acid and diluted to ca. 10% AcOH (1 ml pr well) and transferred to individual glass vials and freeze dried. The peptides were dissolved in buffer: 50 mM sodium phosphate, 70 mM sodium chloride, 0.05% Tween 80, pH 7.4 or 20 mM HEPES, 2.2% glycerol, 0.05% Polysorbate 80, pH 6.5 and handed over to PK *in vivo* (see main text)

TAMRA-labelled peptides. Peptides for the FP assays were synthesized on a 10 µmol scale in 96 plate format using 20 mg Fmoc-PAL-PS resin. The procedure for the peptides were as described above. The fluorophore TAMRA was added to the side chain of lysine after deprotection of ivDde protecting group by 2% hydrazine in DMF for 30 min. TAMRA (2 eq) was preactivated by PyAOP (2 eq) and DIPEA (4 eq) in DMF and added to the peptidyl-resin. After 1 hour this procedure was repeated and the second coupling allowed to couple overnight. TAMRA labelled peptides were cleaved and HPLC purified as the above described method for individual peptides. The peptides were cleaved and deprotected with TFA/TIPS/thioanisole (95:2.5:2.5) for 3 h and precipitated with diethyl ether. After washing with diethyl ether 5 times through a 0.45 µm filter (solvinert plates Millipore), the peptides were dried and purified as described above.

**Table S1**. Purity (%), retention time (ret. time) and found mass (average) of peptides. See Table 2 for details concerning UPLC conditions a-e.

| **No.** | **Peptide** | **Purity  (%)** | **Retention time** | **Theoretical mass (average)** | **Mass found (average)** |
| --- | --- | --- | --- | --- | --- |
|  | PYY_1-36_ | 97.0 | 3.4^e^ | 4309.8 | 4309.0 |
|  | PYY_3-36_ | 98.0 | 5.8^b^ | 4049.5 | 4050.0 |
|  | PP_1-36_ | 96.7 | 2.5^f^ | 4181.7 | 4180.9 |
| **1** | [(C18diacid-γGlu-2xAdo)]PYY_3-36_ | 94.8 | 7.6^a^ | 4765.3 | 4764.4 |
| **2** | [K^ε4^(C18diacid-γGlu-2xAdo)]PYY_3-36_ | 95.8 | 7.9^a^ | 4765.3 | 4764.9 |
| **3** | [K^ε5^(C18diacid-γGlu-2xAdo)]PYY_3-36_ | 95.7 | 7.2^a^ | 4796.4 | 4796.0 |
| **4** | [K^ε6^(C18diacid-γGlu-2xAdo)]PYY_3-36_ | 92.0 | 7.2^a^ | 4764.4 | 4764.0 |
| **5** | [K^ε7^(C18diacid-γGlu-2xAdo)]PYY_3-36_ | 98.1 | 8.0^a^ | 4822.4 | 4822.0 |
| **6** | [K^ε8^(C18diacid-γGlu-2xAdo)]PYY_3-36_ | 98.0 | 7.7^a^ | 4796.4 | 4797.0 |
| **7** | [K^ε9^(C18diacid-γGlu-2xAdo)]PYY_3-36_ | 95.0 | 7.1^a^ | 4836.5 | 4836.0 |
| **8** | [K^ε10^(C18diacid-γGlu-2xAdo)]PYY_3-36_ | 96.0 | 8.2^a^ | 4764.4 | 4765.0 |
| **9** | [K^ε11^(C18diacid-γGlu-2xAdo)]PYY_3-36_ | 94.0 | 7.7^a^ | 4778.4 | 4777.4 |
| **10** | [K^ε12^(C18diacid-γGlu-2xAdo)]PYY_3-36_ | 92.3 | 8.0^a^ | 4822.4 | 4822.5 |
| **11** | [K^ε13^(C18diacid-γGlu-2xAdo)]PYY_3-36_ | 93.5 | 7.9^a^ | 4806.4 | 4806.6 |
| **12** | [K^ε14^(C18diacid-γGlu-2xAdo)]PYY_3-36_ | >98 | 7.5^a^ | 4796.4 | 4796.0 |
| **13** | [K^ε15^(C18diacid-γGlu-2xAdo)]PYY_3-36_ | >98 | 7.4^a^ | 4764.4 | 4765.0 |
| **14** | [K^ε16^(C18diacid-γGlu-2xAdo)]PYY_3-36_ | 98.4 | 7.4^a^ | 4764.4 | 4765.0 |
| **15** | [K^ε17^(C18diacid-γGlu-2xAdo)]PYY_3-36_ | 94.7 | 7.5^a^ | 4780.4 | 4780.0 |
| **16** | [K^ε18^(C18diacid-γGlu-2xAdo)]PYY_3-36_ | 94.7 | 7.5^a^ | 4779.4 | 4780.3 |
| **17** | [K^ε19^(C18diacid-γGlu-2xAdo)]PYY_3-36_ | 94.0 | 7.8^a^ | 4737.3 | 4737.2 |
| **18** | [K^ε20^(C18diacid-γGlu-2xAdo)]PYY_3-36_ | >98 | 7.7^a^ | 4730.3 | 4731.0 |
| **19** | [K^ε21^(C18diacid-γGlu-2xAdo)]PYY_3-36_ | 93 | 7.5^a^ | 4730.3 | 4729.3 |
| **20** | [K^ε22^(C18diacid-γGlu-2xAdo)]PYY_3-36_ | 95.0 | 7.6^a^ | 4822.4 | 4822.3 |
| **21** | [K^ε23^(C18diacid-γGlu-2xAdo)]PYY_3-36_ | >98 | 7.6^a^ | 4806.4 | 4805.6 |
| **22** | [K^ε24^(C18diacid-γGlu-2xAdo)]PYY_3-36_ | 97 | 7.3^a^ | 4780.4 | 4780.0 |
| **23** | [K^ε25^(C18diacid-γGlu-2xAdo)]PYY_3-36_ | >98 | 7.4^a^ | 4737.3 | 4736.4 |
| **24** | [K^ε26^(C18diacid-γGlu-2xAdo)]PYY_3-36_ | 97 | 7.2^a^ | 4756.4 | 4755.5 |
| **25** | [K^ε27^(C18diacid-γGlu-2xAdo)]PYY_3-36_ | >98 | 7.3^a^ | 4730.3 | 4730.0 |
| **26** | [K^ε28^(C18diacid-γGlu-2xAdo)]PYY_3-36_ | >98 | 7.5^a^ | 4780.4 | 4780.0 |
| **27** | [K^ε29^(C18diacid-γGlu-2xAdo)]PYY_3-36_ | 95.0 | 8.2^a^ | 4779.4 | 4779.0 |
| **28** | [K^ε30^(C18diacid-γGlu-2xAdo)]PYY_3-36_ | 96.0 | 8.1^a^ | 4780.4 | 4779.9 |
| **29** | [K^ε31^(C18diacid-γGlu-2xAdo)]PYY_3-36_ | 96.2 | 7.8^a^ | 4794.4 | 4794.0 |
| **30** | [K^ε32^(C18diacid-γGlu-2xAdo)]PYY_3-36_ | >98 | 7.8^a^ | 4792.4 | 4791.5 |
| **34** | [K^ε30^(C14diacid-γGlu-2xAdo)]PYY_3-36_ | 94.3 | 7.3^a^ | 4724.2 | 4724.1 |
| **35** | K^ε30^(C16diacid-γGlu-2xAdo)]PYY_3-36_ | 96.0 | 7.5^a^ | 4752.3 | 4752.0 |
| **36** | K^ε30^(C20diacid-γGlu-2xAdo)]PYY_3-36_ | 96.0 | 8.5^a^ | 4808.4 | 4809.0 |
| **37** | [K^ε30^(C14diacid-2xAdo)]PYY_3-36_ | 97.0 | 6.8^a^ | 4595.1 | 4592.0 |
| **38** | [K^ε30^(C16diacid-2xAdo)]PYY_3-36_ | 97.8 | 7.2^a^ | 4623.2 | 4622.9 |
| **39** | [K^ε30^(C18diacid-2xAdo)]PYY_3-36_ | >98 | 7.8^a^ | 4651.2 | 4650.3 |
| **40** | [K^ε30^(C20diacid-2xAdo)]PYY_3-36_ | >98 | 8.2^a^ | 4679.3 | 4678.4 |
| **41** | [K^ε30^(C18diacid-γGlu)]PYY_3-36_ | 94.0 | 9.0^a^ | 4490.0 | 4490.0 |
| **42** | [K^ε30^(C18diacid-γGlu-4xAdo)]PYY_3-36_ | 97.6 | 7.8^a^ | 5070.7 | 5070.0 |
| **43** | [K^ε30^(C18diacid-γGlu-6xAdo)]PYY_3-36_ | 93.0 | 7.5^a^ | 5361.0 | 5361.9 |
| **44** | [K^ε30^(C16-γGlu-2xAdo)]PYY_3-36_ | 97.0 | 8.7^a^ | 4722.3 | 4721.7 |
| **45** | [K^ε4^(C18diacid-γGlu-2xAdo),MeArg^35^]PYY_3-36_ | 97.6 | 3.2^f^ | 4779.4 | 4778.8 |
| **46** | [Ala^4^,K^ε30^(C18diacid-γGlu-2xAdo)]PYY_3-36_ | >99.0 | 7.5^a^ | 4723.3 | 4722.5 |
| **47** | [Arg^4^,K^ε30^(C18diacid-γGlu-2xAdo)]PYY_3-36_ | 97.0 | 7.3^a^ | 4808.4 | 4808.0 |
| **48** | [Asp^18^,K^ε30^(C18diacid-γGlu-2xAdo)]PYY_3-36_ | 96.0 | 9.1^a^ | 4781.4 | 4780.5 |
| **49** | [Ac,Ala^4^,K^ε30^(C18diacid-γGlu-2xAdo)]PYY_3-36_ | 97.0 | 4.5^c^ | 4765.3 | 4764.4 |
| **50** | [Ac, Arg^4^,K^ε30^(C18diacid-γGlu-2xAdo)]PYY_3-36_ | 97.0 | 7.6^a^ | 4850.4 | 4850.4 |
| **51** | [Ac, Ala^4^, Asp^18^,K^ε30^(C18diacid-γGlu-2xAdo)]PYY_3-36_ | >99 | 7.8^a^ | 4766.3 | 4765.4 |
| **52** | [Ac, Arg^4^, Gln^18^,K^ε30^(C18diacid-γGlu-2xAdo)]PYY_3-36_ | 95.3 | 3.2^d^ | 4822.4 | 4821.9 |
| **53** | [K^ε4^(TAMRA)K^ε7^(C18diacid-γGlu-2xAdo)]PYY_3-36_ | 95.0 | 8.4^b^ | 5236.9 | 5235.0 |
| **54** | [K^ε4^(TAMRA)K^ε10^(C18diacid-γGlu-2xAdo)]PYY_3-36_ | 98.5 | 8.4^b^ | 5177.8 | 5176.9 |
| **55** | [K^ε4^(TAMRA)K^ε19^(C18diacid-γGlu-2xAdo)]PYY_3-36_ | 95.4 | 8.5^b^ | 5150.8 | 5150.8 |
| **56** | [K^ε4^(TAMRA)K^ε25^(C18diacid-γGlu-2xAdo)]PYY_3-36_ | 97.8 | 8.6^b^ | 5150.8 | 5150.8 |
| **57** | [K^ε4^(TAMRA)K^ε30^(C18diacid-γGlu-2xAdo)]PYY_3-36_ | 96.5 | 8.3^b^ | 5193.8 | 5193.9 |
| **58** | [K^ε4^(TAMRA)K^ε33^(C18diacid-γGlu-2xAdo)]PYY_3-36_ | 91.1 | 8.3^b^ | 5150.8 | 5149.9 |
| **59** | [K^ε4^(TAMRA)K^ε30^(C14diacid-γGlu-2xAdo)]PYY_3-36_ | 98.5 | 7.5^b^ | 5137.7 | 5136.8 |
| **60** | [K^ε4^(TAMRA)K^ε30^(C16diacid-γGlu-2xAdo)]PYY_3-36_ | 99.2 | 7.9^b^ | 5165.7 | 5164.8 |
| **61** | [K^ε4^(TAMRA)K^ε30^(C20diacid-γGlu-2xAdo)]PYY_3-36_ | 97.9 | 8.8^b^ | 5221.8 | 5220.9 |

**Table S2**. UPLC gradients used in analysis of peptides. The method number (a-g) in column 1 is applied to Table 1 in column “Ret. time).

| **Method** | **Column** (40 ^°^C) | **Flow rate** | **Gradient**  (A buffer H_2_O + 0,05% TFA) (B buffer ACN + 0.05% TFA) |
| --- | --- | --- | --- |
| a | Waters BEH C18 1,7 μm; 2.1 mm x 150 mm | 0.40 ml/min | 5% buffer B to 60% buffer B in 16 min. |
| b | Waters BEH C18 1,7 μm; 2.1 mm x 150 mm | 0.40 ml/min | 5% buffer B to 95% buffer B in 16 min. |
| c | Waters BEH C18 1,7 μm; 2.1 mm x 150 mm | 0.40 ml/min | 5% buffer B to 60% buffer B in 10 min. |
| d | Waters BEH C18 1,7 μm; 2.1 mm x 50 mm | 0.45 ml/min | 5% buffer B to 60% buffer B in 6 min |
| e | Waters BEH C18 1,7 μm; 2.1 mm x 50 mm | 0.45 ml/min | 15% buffer B to 35% buffer B in 3.5 min |
| f | Waters BEH C18 1,7 μm; 2.1 mm x 50 mm | 0.45 ml/min | 25% buffer B to 45% buffer B in 3.5 min |

**ADMET**

Experimental: Plasma samples were analyzed by LC-MS on an LTQ-Orbitrap (ThermoFisher Scientiifc, Bremen) to which Accela HPLC pumps and an autosampler were connected (both from ThermoFisher). The mass spectrometer was equipped with an electrospray interface, which was operated in positive ionisation mode. Analysis was conducted in a full scan mode from m/z 300-1800. HPLC was performed on a Jupiter Proteo column (4µ) 90A (50 x 2.0 mm ID). Mobile phases consisted of A. 0.1% formic acid and B. 0.1% formic acid in acetonitrile. A gradient was run from 5% B to 60% B over 10min. The flow rate was 0.3 ml/min. For analysis of plasma samples, 30 µl plasma was precipitated with 90 µl of ethanol and after centrifugation, the supernatant was diluted with one volume of water prior to LC/MS analysis.

_
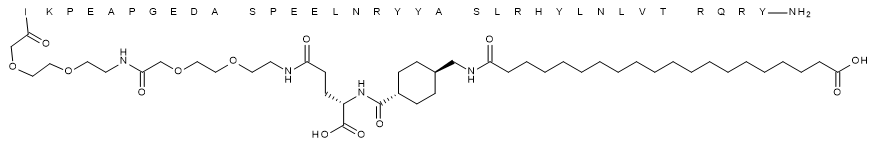
_

**Figure S1**. Fatty acid acylated PYY_3-34_ metabolite identified as the major metabolite in minipig when dosed with [C20diacid-Trx-γGlu-2xAdo]PYY_3-36_. Time profiles were obtained by LC/MS (see below) of pooled plasma samples (n=2).

**Figure S2**

MS data of [C20diacid-Trx-γGlu-2xAdo]PYY_3-36_ dosed to mini-pigs. MS spectra were averaged from 7.6-8.2 min from LC/MS analysis, where parent PYY analogue and the two metabolites eluted. Identity of the major metabolite, [C20diacid-Trx-LgGly-2xAdo]PYY3-34 was confirmed by MS/MS (not shown). All metabolites were identified with a mass accuracy +/- 3ppm.


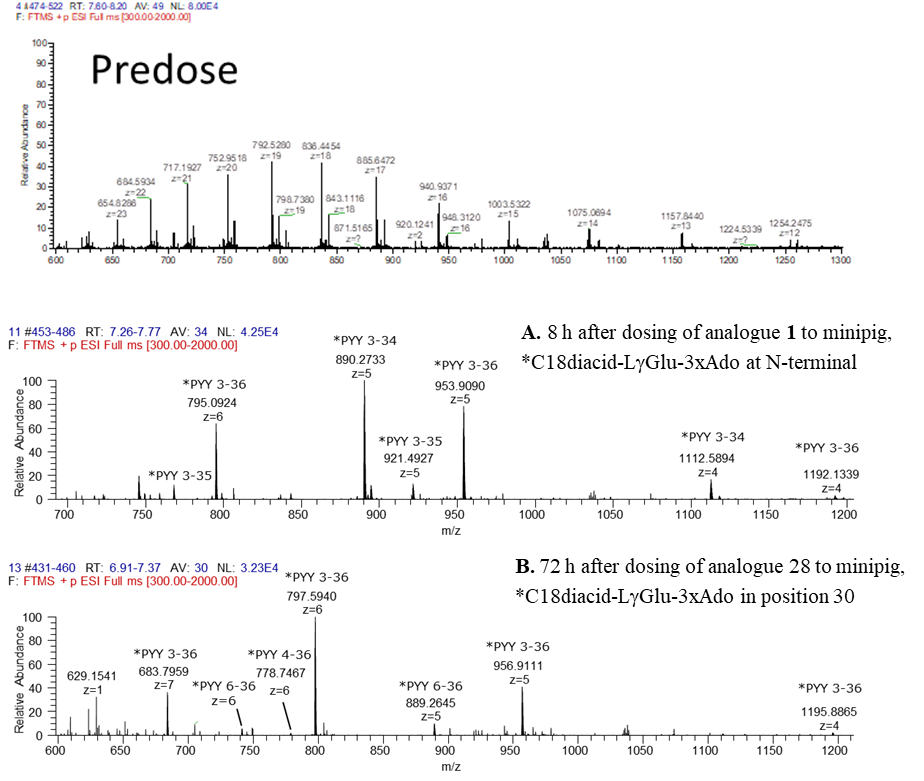


**Figure S3.** Identification of *in vivo* degradation products following i.v. dosing to minipigs (50 nmol/kg, n=2) of A. analogue **1** (8h after dosing) and B. Analogue **28** (72h after dosing). Analogue **1** was lipidated with C18diacid-γGlu-2xAdo in the N-terminal and analogue **28** was lipidated in position 30. All measured monoisotopic masses from LC/MS analysis were in accordance with the theoretical masses (mass accuracy within +/- 3ppm). m/z, mass-to-charge state; z, charge state.


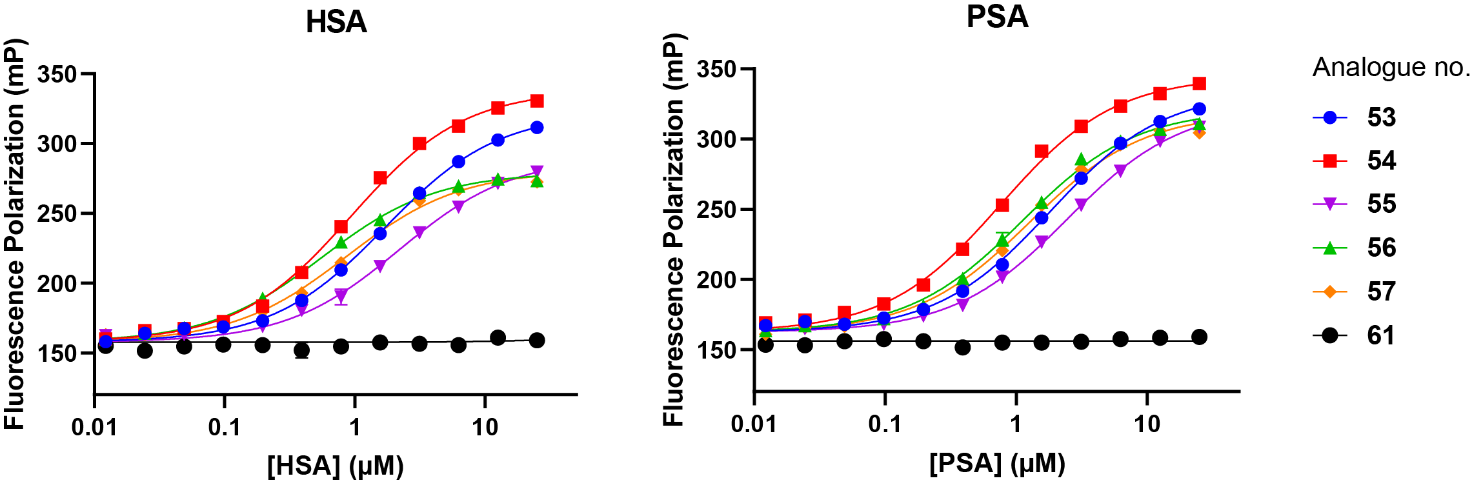


**Figure S4**. Representative data from fluorescence polarization experiments. The data is fitted with a simple binding isotherm. TAMRA-labelled PYY without a fatty acid was included as a control and does not show any binding to human (HSA) or porcine (PSA) serum albumin.


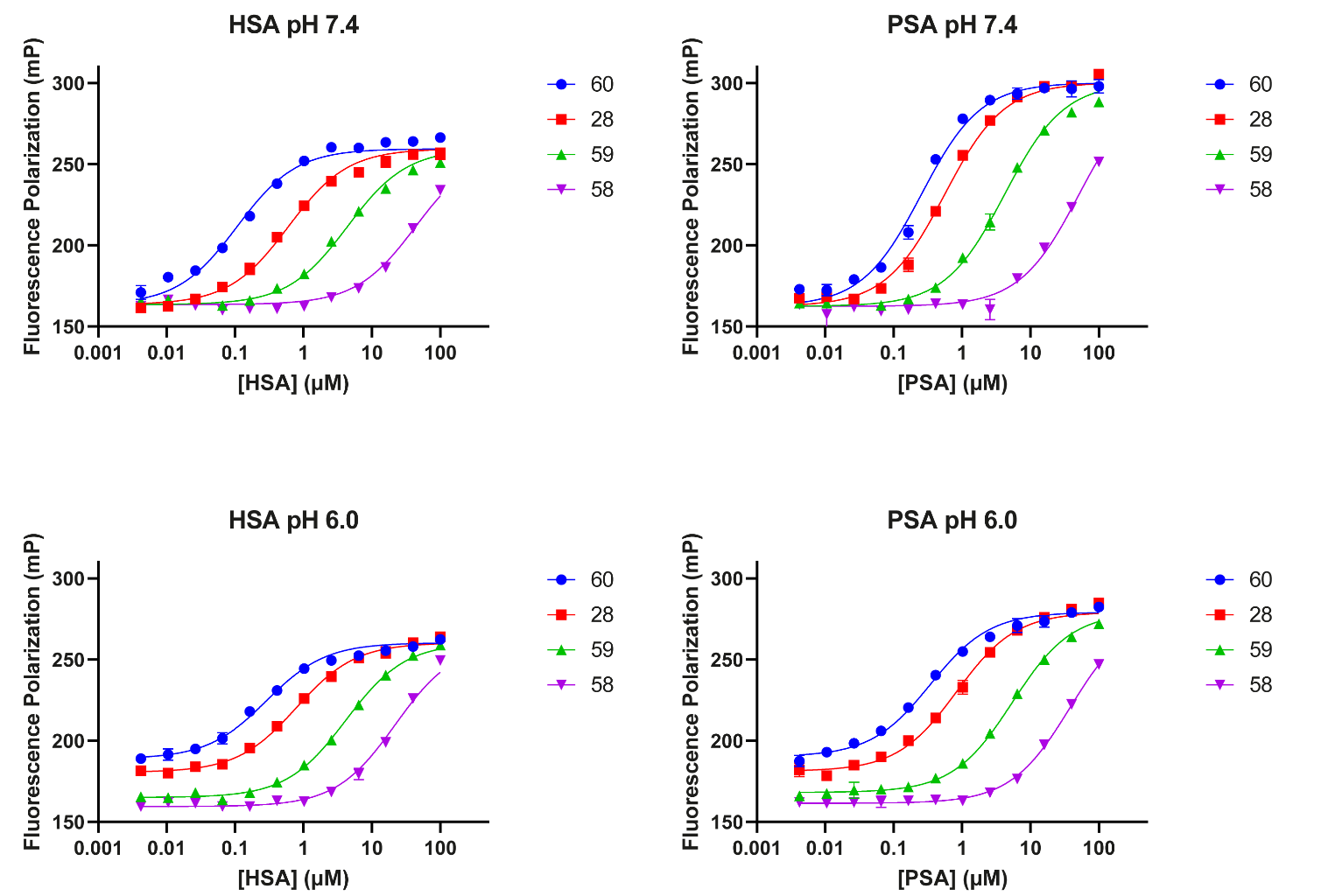


**Figure S5**. Representative data from fluorescence polarization experiments at two different pH values. The data is fitted with a simple binding isotherm. Note that at pH 6.0, peptides 60 and 28 show an increased fluorescence polarization in absence of albumin. This observation is due to increased stickiness of the peptides under these conditions, which leads to a loss of absolute fluorescence intensity and a higher background contribution to the measured polarization signal. Thus, for fitting the pH 7.4 data, both background and maximal amplitude could be shared among the fits to increase robustness, whereas for fitting the pH 6.0 data, only the maximal amplitude is shared.
